# Human Motor Cortical Activity Is Selectively Phase-Entrained on Underlying Rhythms

**DOI:** 10.1371/journal.pcbi.1002655

**Published:** 2012-09-06

**Authors:** Kai J. Miller, Dora Hermes, Christopher J. Honey, Adam O. Hebb, Nick F. Ramsey, Robert T. Knight, Jeffrey G. Ojemann, Eberhard E. Fetz

**Affiliations:** 1 Department of Neurosurgery, Stanford University, Stanford, California, United States of America; 2 Program in Neurobiology and Behavior, University of Washington, Seattle, Washington, United States of America; 3 Department of Physics, University of Washington, Seattle, Washington, United States of America; 4 Department of Neurology and Neurological Sciences, Stanford University, Stanford, California, United States of America; 5 Section Brain Function and Plasticity, Department of Neurology and Neurosurgery, Rudolf Magnus Institute of Neuroscience, University Medical Center Utrecht, Utrecht, The Netherlands; 6 Department of Psychology and Princeton Neuroscience Institute, Princeton University, Princeton, New Jersey, United States of America; 7 Department of Neurological Surgery, University of Washington, Seattle, Washington, United States of America; 8 Helen Wills Neuroscience Institute, University of California Berkeley, Berkeley, California, United States of America; 9 Department of Physiology and Biophysics, University of Washington, Seattle, Washington, United States of America; University of Oxford, United Kingdom

## Abstract

The functional significance of electrical rhythms in the mammalian brain remains uncertain. In the motor cortex, the 12–20 Hz beta rhythm is known to transiently decrease in amplitude during movement, and to be altered in many motor diseases. Here we show that the activity of neuronal populations is phase-coupled with the beta rhythm on rapid timescales, and describe how the strength of this relation changes with movement. To investigate the relationship of the beta rhythm to neuronal dynamics, we measured local cortical activity using arrays of subdural electrocorticographic (ECoG) electrodes in human patients performing simple movement tasks. In addition to rhythmic brain processes, ECoG potentials also reveal a spectrally broadband motif that reflects the aggregate neural population activity beneath each electrode. During movement, the amplitude of this broadband motif follows the dynamics of individual fingers, with somatotopically specific responses for different fingers at different sites on the pre-central gyrus. The 12–20 Hz beta rhythm, in contrast, is widespread as well as spatially coherent within sulcal boundaries and decreases in amplitude across the pre- and post-central gyri in a diffuse manner that is not finger-specific. We find that the amplitude of this broadband motif is entrained on the phase of the beta rhythm, as well as rhythms at other frequencies, in peri-central cortex during fixation. During finger movement, the beta phase-entrainment is diminished or eliminated. We suggest that the beta rhythm may be more than a resting rhythm, and that this entrainment may reflect a suppressive mechanism for actively gating motor function.

## Author Summary

We have long known that there are rhythmic oscillations in the mammalian brain. Although the power in these rhythms changes during behavior, their relevance for brain function has been something of a mystery. In this study, the particular role of rhythms in human motor cortex was studied both during rest and during engagement in different finger movements. Arrays of electrocorticographic electrodes were placed directly on frontal, temporal, and parietal brain surfaces to measure the electrical potential. These measurements reveal that movement of different fingers produces spatially focal and finger-specific changes in local neuronal population activity, along with spatially diffuse and finger-nonspecific decreases in 12–20 Hz β-rhythm power. Apart from these power changes, there are independent interactions between the β-rhythm and local cortical activity. During rest, 5–20% of the variation in local cortical activity is due to entrainment on the phase of the β-rhythm over the pre- and post-central gyri, but the interaction is significantly diminished during movement. This shifting entrainment suggests that the β-rhythm is not simply a background process that is suppressed during movement, but rather that the β-rhythm plays an active and important role in motor processing.

## Introduction

**Figure 1:**
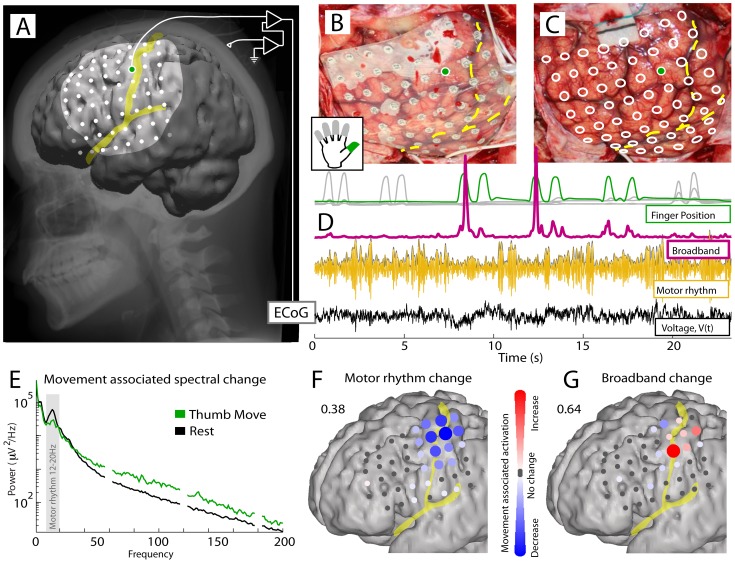
Electrocorticographic (ECoG) activity in rolandic cortex during movement and rest (Subject 1). (**A**) ECoG potential is measured from the brain surface. (**B**) ECoG electrodes in situ, embedded in silastic. (**C**) Electrode positions on the cortical surface. (**D**) Traces show simultaneous finger position color-coded as in inset (top) along with aspects of the potential timeseries. The raw ECoG voltage at bottom (black) is shown from the M1 site marked with green dot in A–C. “Motor rhythm” is 12–20 Hz bandpassed ECoG (gold trace). “Broadband spectral change” (pink) is the timeseries of an estimate of the coefficient 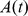 in a power law in the power spectral density of the form 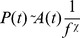. (**E**) The power spectral density during movement (green) and rest (black) reveals a decrease in a peaked process at low frequencies (gray – 12–20 Hz), and a broadband increase across the rest of the frequency range during movement (60 Hz line noise and harmonics omitted). (**F**) The spatial distribution of sites showing a decrease in 12–20 Hz power associated with thumb movement. (color represents a signed r^2^ measurement, scaled to the maximum across the array: 0.38). In these figures the Central sulcus and Sylvian fissure are shown in yellow. (**G**) Broadband spectral changes associated with thumb movement are similarly shown (maximum: 0.64).

**Figure 2:**
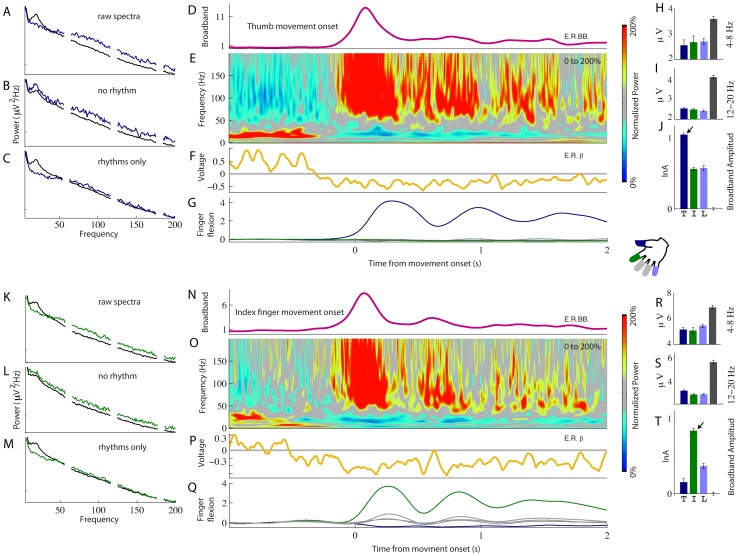
Spectral changes in two adjacent precentral motor cortex sites (1 cm apart; Subject 2). (**A**) Power spectral density (PSD) of the ECoG potential during epochs of thumb movement (blue) and rest (black). (**B**) The PSD re-constructed without the 2^nd^–4^th^ principal spectral components (PSCs). (**C**) The PSD reconstructed with the 2^nd^–4^th^ PSCs only. (**D**) Timecourse of broadband spectral change (pink), as in (B) averaged with respect to onset of the first cued thumb movement at time zero. (**E**) Average time-varying PSD. (**F**) Average time-varying envelope of 12–20 Hz filtered voltage (gold - “motor rhythm”). (**G**) Average finger flexion, timed to onset of thumb movement (thumb flexion – blue; index – green; others - gray). (**H–J**) Average 4–8 Hz amplitude (H), 12–20 Hz amplitude (I), and broadband change (J), during epochs of movement of thumb (dark blue, “T” at bottom), index (green, “I”), and little finger (light blue, “L”), as well as rest (dark gray). Right-most error bars in (J) are for rest epochs; the task epochs are zeroed with respect to mean of rest epochs. Arrow pointing to the tall bar denotes thumb specificity. (**K–T**) As in (A–J), but in an adjacent site 1 cm away, showing changes related to index finger movement.

Human motor behaviors such as reaching, grasping and speaking, are executed and controlled by the somatomotor regions of the cerebral cortex, which are located immediately anterior and posterior of the central sulcus. This peri-central system is known to manifest a 12–20 Hz electrical oscillation known as the beta rhythm [1], [2], which has long been known to have an inverse relation to sensory processing [3] and motor production [4], [5], [6]. This beta rhythm is decreased during movement initiation and production [7], [8], [9], [10] (illustrated in Figures 1&2), and also during motor imagery [11]. Following cessation of movement, there is a rebound augmentation in beta amplitude, specific to peri-central somatomotor and somatosensory cortex [12], [13]. With movement disorders, variations in the amplitude of the brain surface beta rhythm are differentially associated with specific motor diseases [14].

Classically, diseases in the brain have been approached in terms of local pathology. For example, a stroke that abolishes the blood supply to the precentral gyrus, or a precentral tumor can produce paralysis [15]. However, we are beginning to understand that some are diseases of brain dynamics, which involve functionally specific brain areas, but are ultimately a result of dysfunctional interactions. Dysfunctional inter-regional dynamics have been explored using measures of cortical metabolic activity (fMRI and PET), in studies of epilepsy [16], Alzheimer's disease [17], depression and other neuropsychiatric disorders [18], [19], and, most relevant for the present study, motor disease [20], [21], [22]. However, these measures of brain metabolism are tied to metabolic timescales (e.g. at least 5–10 s); in contrast, the computational neural network dynamics occur at the faster timescales over which cognition and behavior are modulated (50–250 ms). Aspects of macroscale and network physiology at these timescales in the brain can now be captured by changes in brain surface electrical potentials.

Despite the association between the beta rhythm and motor function, it is not known whether the rhythm plays an active role in altering the computations taking place in somatomotor cortex, or whether it is epiphenomenon of cortical state changes determined by other mechanisms. Here we present new evidence for the role of the beta rhythm in organizing somatomotor function by quantifying the relation between rhythmic population activity on the time-scales of tens of milliseconds in sites across the lateral cerebral cortex.

**Figure 3:**
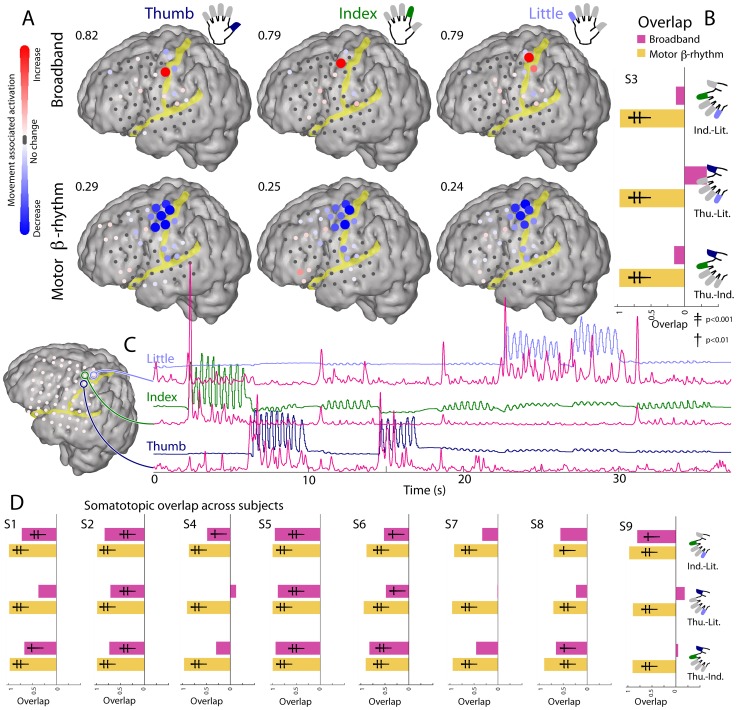
ECoG broadband power resolves somatotopic representation of fingers (Subject 3). (**A**) Changes in broadband and 12–20 Hz beta power at different cortical sites for movement of thumb, index, and little finger. Colors denote a signed r^2^ measurement of increases and decreases in power with movement relative to rest (individually scaled with maximum to upper left of each plot). (**B**) Quantification of spatial overlap between changes associated with finger-movements. (1 is maximum possible overlap and 0 is no overlap; negative overlaps occur when increases overlap with decrease). Resampling significance, single cross denotes p<0.01, double denotes p<0.001, that the overlap happened by chance. (**C**) Traces of thumb (dark blue), index (green), and little finger (light blue) position, with corresponding timecourse of broadband spectral change (pink; approximated by the projection of the 1^st^ PSC to the wavelet-obtained dynamic spectrum) for 3 cortical sites. (**D**) Quantification of overlap in finger movement activation for 8 other subjects (denoted “S#”).

Recent work has shown that the phase of the beta rhythm in the local field potential is correlated with the firing time of individual neurons in primary motor cortex [23], [24], [25]. Here we use subdural electrocorticography (ECoG) at the brain surface to examine this relationship for neural population activity (Figure 1). From these ECoG recordings, a correlate of mean instantaneous firing rate across the population of neurons beneath the electrode can be extracted in the form of broadband spectral power [26], [27], [28], [29]. Changes in this extracted broadband activity have been demonstrated to capture movement-related and visual cortical changes at the 50–100 ms timescales on which the faster aspects of cortical processing take place [30], [31] (Figures 2,3).

In this study, we examined the properties of the beta rhythm at rest during simple visual fixation and during a basic finger movement task. By documenting how broadband spectral changes vary with the phase of underlying motor beta rhythm, we show that local cortical activity has a robust entrainment on the phase of the beta rhythm, specifically in peri-central motor areas. This relation of the beta rhythm to local neuronal activity is a property of the “resting brain”: it is present during fixation and when resting, and is selectively diminished during movement, along with the amplitude of the beta rhythm. We hypothesize that this phenomenon, here observed for the peri-central beta rhythm, may be one example of a general motif for cortical-subcortical circuits, and that this motif will be observed with different brain rhythms across cortical areas and behavioral states.

## Results

### Movement-related spectral changes

Results were obtained from ECoG recordings in 14 human subjects (see Methods). As shown in Figure 1E, the averaged power spectral density (PSD) of the ECoG potential during movement and rest exhibits a general broadband 1/f^χ^ shape, with superimposed rhythms that deviate from this 1/f^χ^ structure at particular low frequencies [28], [31]. During motor behavior, these averaged PSDs commonly reveal decreases in power in the beta rhythm, and an increase in the broadband power (Figures 1E, 2A&J) [8], [9]. The spatial distribution of the sites showing beta rhythm change is relatively large, covering pre- and post-central cortex (Figure 1F). In contrast, the broadband spectral power changes are localized to specific sites representing the digit that is moved (Figure 1G).

To illustrate the temporal and spatial specificity of the broadband power, Figure 2 shows the spectral changes for two finger movements observed at two adjacent cortical sites. During thumb movement (top half) the PSD shows the increase in broadband power and decrease in beta rhythms (Figure 2A). The PSD can be naively decoupled using a variant of principal component analysis (Methods) to show how the powers in different frequency bands vary with respect to one another. The spectra during movement and rest are reconstructed without the 2^nd^–4^th^ principal spectral components (PSCs) in Figure 2B. This reconstruction shows that during movement power increases across all frequencies, including low frequencies. Conversely, the spectrum reconstructed for only the 2^nd^–4^th^ PSCs (Figure 2C) shows that the change is specific to narrowband frequencies in the β-rhythm range. Thus, changes in the power spectrum of the ECoG potential can be understood as a combination of a band-limited (rhythmic) component and a broadband (non-rhythmic) component.

The time-course of spectral changes relative to thumb movement are shown in averages aligned with movement onset (Figure 2D–G). A time-varying estimate of broadband spectral change was obtained by projecting the PSD at each time point onto the coefficient vector of the first principal spectral component (Figures 2, 3). The resulting pattern of broadband change over time approximates the time-varying amplitude function 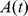 in the power-law relationship: 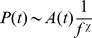. Broadband spectral change was isolated from the continuous dynamic spectrum, applied to only the 1st PSC, and averaged with respect to the first thumb movement following each cue (Figure 2D). Note that broadband power begins to change 50–100 ms prior to the onset of thumb movement. The average time-varying PSD, scaled as a percentage of mean power at each frequency (Figure 2E) shows the inverse relation of beta and high frequency power as a function of time relative to movement. Note that the change in β activity begins prior to the broadband spectral change. The drop in beta power with movement is illustrated in Figure 2F. The specificity of this site for thumb movements is shown by the greater increase in broadband power compared to movement of other digits (Figure 2J). In contrast, all digit movements were associated with a similar drop in beta power relative to rest (Figure 2H,I). The lower half of Figure 2 documents similar results for movement of the index finger in recordings from an adjacent site, separated by 1 cm from the thumb site.

While the first movement in a set is sometimes associated with the largest amount of cortical change, this was not reliably the case, likely because movement frequency varied across epochs within and across subjects. Emerging work, using primary motor and somatosensory microECoG arrays during supervised pacing of repeated finger movement, shows that cortical activity attenuation with repeated movement is robust, and is a function of movement rate [32].

### Somatotopy of movement-related spectral changes

Figure 3 demonstrates that, when examined across the cortex, broadband spectral changes during movement are highly localized and somatotopically distinct for different fingers (A). In contrast, changes in beta power are broadly distributed and essentially the same for any finger movement. The instantaneous broadband power shows a clear temporal correlation with individual finger movements (C); note that the broadband trace obtained at each site correlates with the position of only one of the fingers and not the others. The spatial overlap of changes for particular pairs of digits is summarized in Figure 3B. Overlap (B) is quantified by the proportion of sites showing similar changes with movement of the two digits (Methods), and is larger for the beta power (near complete overlap) than broadband power (largely distinct spatially). Similar overlap patterns are documented for the other eight subjects as well (D). Broadband spectral changes show largely non-overlapping representation between digits, while the distribution of change in the beta rhythm overlaps almost completely in all cases. Digit-specific electrodes were typically 1 cm from one another (also see Figures S5, S6, S7, S8, S9, S10 in Text S1).

### Coupling between low frequency rhythm phase and amplitude of broadband spectral change

**Figure 4:**
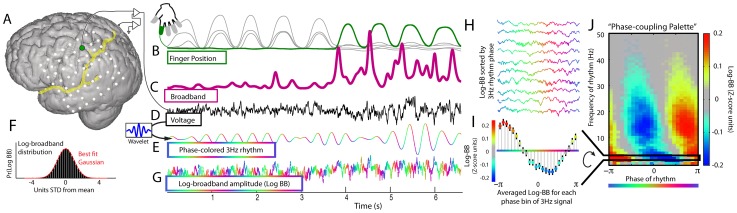
Relation between broadband power and phase of ECoG rhythms (subject 4). (**A**) ECoG potential (in D) was measured from a pre-central motor cortex site (green dot). (**B**) Periodic flexion of index finger (green) and other fingers (gray). The entire movement and rest periods are all examined. (**C**) Fluctuations in broadband power (projection of the 1^st^ PSC, here shown smoothed), extracted from ECoG potential. (**D**) The raw ECoG potential. (**E**) Example low frequency rhythm obtained by convolving ECoG with a 3 Hz wavelet (inset). The resulting time-series is color coded for instantaneous phase (relative to positivity peak of the potential). (**F**) Log values of the time-dependent broadband have a normal distribution. (**G**) The timeseries of the log of the broadband, color coded by the coincident phase of the low frequency 3 Hz rhythm. (**H**) The log-broadband signal is aligned with the phase of the low frequency rhythm (as color-coded). (**I**) The average of the log-broadband amplitude is obtained for phase bins. Error bars denote 3 times the standard error of the mean (3*SEM) for each phase bin. This can be appreciated in one dimension as the “3 Hz coupling row” in G. (**J**) The “Phase coupling palette” obtained by repeating the process detailed in E–I at each frequency from 1–50 Hz., showing modulation of broadband power with the full range of frequencies.

The relation between the instantaneous broadband power and phase of ECoG signal at different frequencies is documented in Figure 4. The analysis is based on recordings obtained during finger movements and rest (B). The dynamic broadband (C) obtained from the raw ECoG potential (D) shows distinct fluctuations related to index finger movement. The ECoG can be filtered with a simple wavelet to obtain a temporally varying estimate of the narrow band-passed signal, as shown for the 3 Hz rhythm in (E). The phase of this signal can be used to extract associated snippets of the broadband signal (H) and obtain a phase-triggered average of the broadband power (I). Repeating this process for all frequencies between 1 and 50 Hz yields the “Phase coupling palette” shown in Figure 4J, which documents the phase relation for all frequencies. The predominant pattern for the range 10–30 Hz shows a tendency for brain activity, as measured by broadband power, to increase just prior to the surface-negative phase (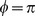) and decrease just prior to the surface positive phase (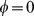) of the beta rhythm. This modulation of broadband amplitude by rhythm phase is also called “rhythmic entrainment”, “phase-amplitude coupling”, and “nested oscillation” in this and other manuscripts [27], [33], [34]. It is important to note that, for broadband coupling to low frequency rhythm, the portions of the broadband that contribute to the coupling measurement will be those with amplitudes that can track the low frequency phase (e.g. for a 10 Hz rhythm, only the portions of broadband >20 Hz will be contributing to the coupling measure between the two).

**Figure 5:**
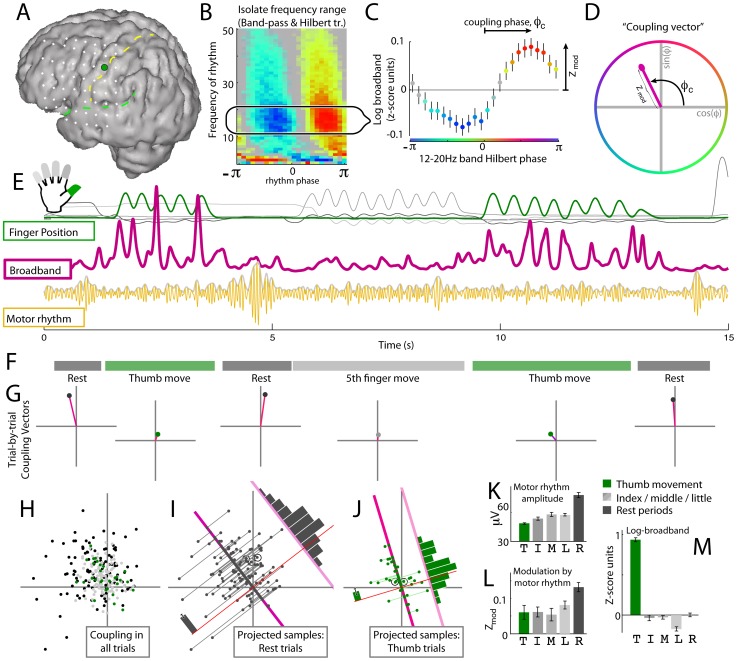
The “coupling vector” and trial-by-trial characterization of modulation. (**A**) The selected precentral motor cortex electrode (green, Subject 3). (**B**) The phase-coupling palette for this site identifying range of low-β band frequencies (12–20 Hz). (**C**) Mean amplitude (+/−3*SEM) of broadband power as function of the Hilbert phase (φ) of the band-passed 12–20 Hz signal. (**D**) The complex “coupling vector” reflects the magnitude, Z_mod_, and phase, φ_c_, of the peak modulation of broadband activity with the rhythm in the complex plane. (**E**) Epochs of finger movement and rest (upper traces) with simultaneous broadband power (pink) and β motor rhythm (gold). Green shows index finger position, and gray shows the other fingers. (**F**) Segments of the data traces corresponding to periods of rest (dark gray), thumb movement (green), and other finger movements (light gray). (**G**) From each behavioral epoch, a single trial coupling vector can be obtained. (**H**) Distribution of coupling vectors across all movement epochs. (**I**) Calculation of the mean coupling vector for the rest condition (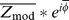). The distribution of coupling values for this behavior is obtained by projecting individual coupling vectors onto the mean complex coupling vector direction (dark pink line). The distribution of these projected values (upper right histogram) gives the error bar indicating 3*SEM for Z_mod_. The corresponding vectors from epochs in G are circled. (**J**) As in (I), for thumb movement epochs. (**K**) Distribution of rhythm amplitudes (envelope of motor rhythm amplitude in E) during epochs of movement and rest. T = Thumb movement epochs, I = Index finger, M = middle finger, L = little finger, R = rest. (**L**) Coupling vector amplitudes (projected as in H and I) for different behavioral types. (**M**) Z-score of log broadband values.

To quantify how rhythmic entrainment with the beta band is modulated with behavior, we summarized the main relationship with a “coupling vector”, as shown in Figure 5. We captured the phase-amplitude coupling across a range of low-β band frequencies (12–20 Hz), rather than at a single frequency, using the Hilbert transform. As described in the Methods, a “coupling vector” in the complex plane (D) succinctly represents the magnitude, Z_mod_, and phase, φ_c_, of the broadband activity phase-coupling with the 12–20 Hz rhythm (C). This coupling vector can be derived for individual behavioral epochs consisting of specific finger movements and rest (E–G), as well as for single trials within epochs (H–J). The global distribution of trial-to-trial coupling vectors across all movement epochs (H) reveals heterogeneous coupling for the different behaviors. A mean coupling vector (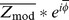) may be obtained for each behavior, and its statistical significance computed as illustrated in (I) for rest and in (J) for thumb movement. The statistics are obtained from the distribution of the projections of individual movement or rest epoch coupling vectors onto the mean vector direction (e.g., upper right histogram in [I]). The Z_mod_ values for the different behaviors (L) show significantly larger entrainment for rest than for any of the movements. A similar difference is seen for the values of the motor rhythm amplitudes (K). The fact that this cortical site (A) is specific for thumb representation is shown by the larger Z-score of log broadband values (M).

### Spatial distribution of phase coupling

**Figure 6:**
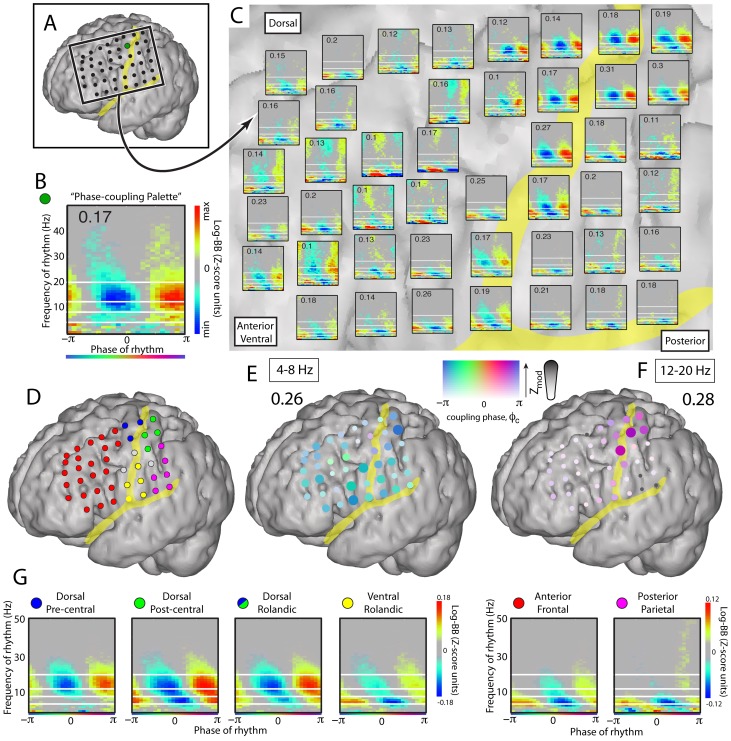
Phase coupling at the lateral cortical surface during baseline fixation task (subject 1). (**A**) Data were obtained from grid of electrodes on the lateral cortical surface. (**B**) The phase coupling palette for the green site in (A). Inset number (0.17) denotes maximum scaling. White lines identify 4, 8, 12, 20 Hz. (**C**) Phase coupling palettes for each cortical site on the grid region in (A), with white lines and scaling maxima noted as in (B). (**D**) Colors of electrode sites delineate cortical regions for average phase palettes in (G). (**E**) 4–8 Hz modulation in the grid using the band-pass & Hilbert transform. The strength of color and diameter denote the magnitude of coupling, and the color denotes the preferred phase of coupling. Note that coupling to the theta range is quite widespread. (**F**) Distribution of 12–20 Hz modulation, as in (E). Note that the modulation is strongest in dorsal peri-central areas. (**G**) Average phase palette by cortical regions delineated in D. The “Dorsal Rolandic” palette combines Dorsal Pre- and Post- central cortex.

**Figure 7:**
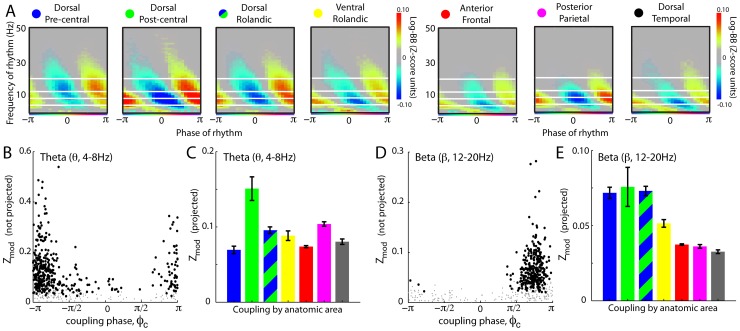
Pooled phase coupling at the lateral cortical surface during baseline fixation task, (subjects 1–4, 7–12). (**A**) Average palette by region, across all subjects. (**B**) Coupling in the 4–8 Hz range. Each dot is an electrode. Black denotes statistically significant coupling. Gray is not significant. (**C**) Average coupling to 4–8 Hz range across all electrodes in each region (color code of bars indicate areas identified in A. Error bars are 95^th^ percentiles obtained by repeated vector projection of only half of electrodes, randomly selected each time). (**D and E**) As in B and C, but for 12–20 Hz.

Similar phase entrainment is present at many cortical sites, as shown in Figure 6 for the baseline fixation task. The phase coupling palettes (C) show distinct differences, with the largest beta rhythm modulation in the dorsal peri-central region (F, G). The average phase palettes computed for different cortical regions (G) show robust phase modulation in the Dorsal Roladic region, with similar but weaker patterns in the Ventral Rolandic, Anterior Frontal and Posterior Parietal sites. The same relationships are seen in the regional palettes combined for nine subjects (Figure 7A). Overall, the 12–20 Hz (β) coupling is strongest peri-centrally, whereas 4–8 Hz (θ) coupling is ubiquitous. The average coupling for each region is summarized by the histograms for the θ and β frequency bands (7C & E, resp.). Note that the preferred phase of coupling is significantly different for 4–8 Hz (θ, preferred phase −0.75π) compared to 12–20 Hz (β, preferred phase 0.81π, significant p<10^6^, by unpaired resampling from electrodes/bands that independently have significant coupling – each at p<0.05 after Bonferroni correction of t-test on projected coupling values for that electrode). When the preferred phase of coupling is compared between brain regions, there are some very weak, but significant, differences, for both a 4–8 Hz and 12–20 Hz range (see Figure S16 in Text S1).

**Figure 8:**
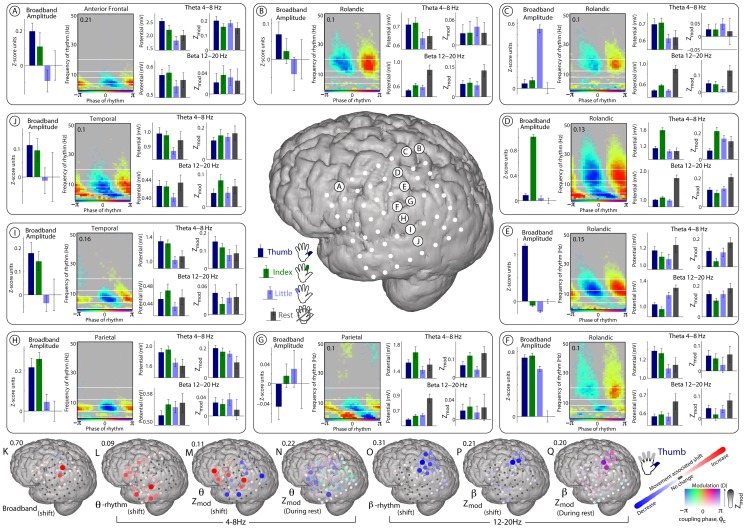
Coupling motifs and movement-associated change in subject 4. (**A**) For the electrode site denoted “A” on the middle cortical rendering. On the far left, the Broadband amplitude (mean +/− 3*SEM) during epochs of movement of the thumb, index, and little fingers, and rest (right-most error bars are for rest, all sub-distributions are zeroed with respect to mean of rest epochs), as in Figure 2J. The coupling palette is as in Figure 4, with the number denoting the maximum scaling for the palette. The top bars to the right of the palette are the amplitude of the 4–8 Hz filtered potential for different behaviors, and the top bars on the far right show the magnitude of coupling during the different behaviors. On the bottom right are filtered amplitudes and the coupling for the 12–20 Hz range. (**B–J**) As in A, but for the electrode sites denoted B–J on the middle cortical rendering. (**K**) The spatial distribution of shift in broadband magnitude during thumb movement compared with rest (a signed r^2^ measurement – maximum scaling denoted by number above the cortical rendering). (**L**) The 4–8 Hz shift in amplitude during thumb movement (also a signed r^2^ measurement). (**M**) The shift in coupling of broadband to 4–8 Hz phase during thumb movement (a signed r^2^ measurement comparing the distributions of projected epoch values, as in Figure 5). (**N**) Coupling of broadband to 4–8 Hz phase during rest epochs. As in Figure 6E, the strength of color as well as electrode diameter denote the magnitude of coupling, and the color itself denotes what the preferred phase of coupling is. In this case, the number above the cortical rendering denotes the maximum Z_mod_ in the array. (**O to Q**) As in L–N, but for 12–20 Hz.

A detailed picture of the phase-coupling motifs and changes with different finger movements for different cortical sites in a representative subject (#4) is shown in Figure 8. Note that the region of strongest absolute coupling with beta during rest epochs runs along the central sulcus. The spatial extent of significant change in coupling (P) lies within both the region of largest coupling during rest (Q) and also within the region of significant change in 12–20 Hz rhythm amplitude (O). The spatial extent of significant broadband change (K) also lies within these regions.

**Figure 9:**
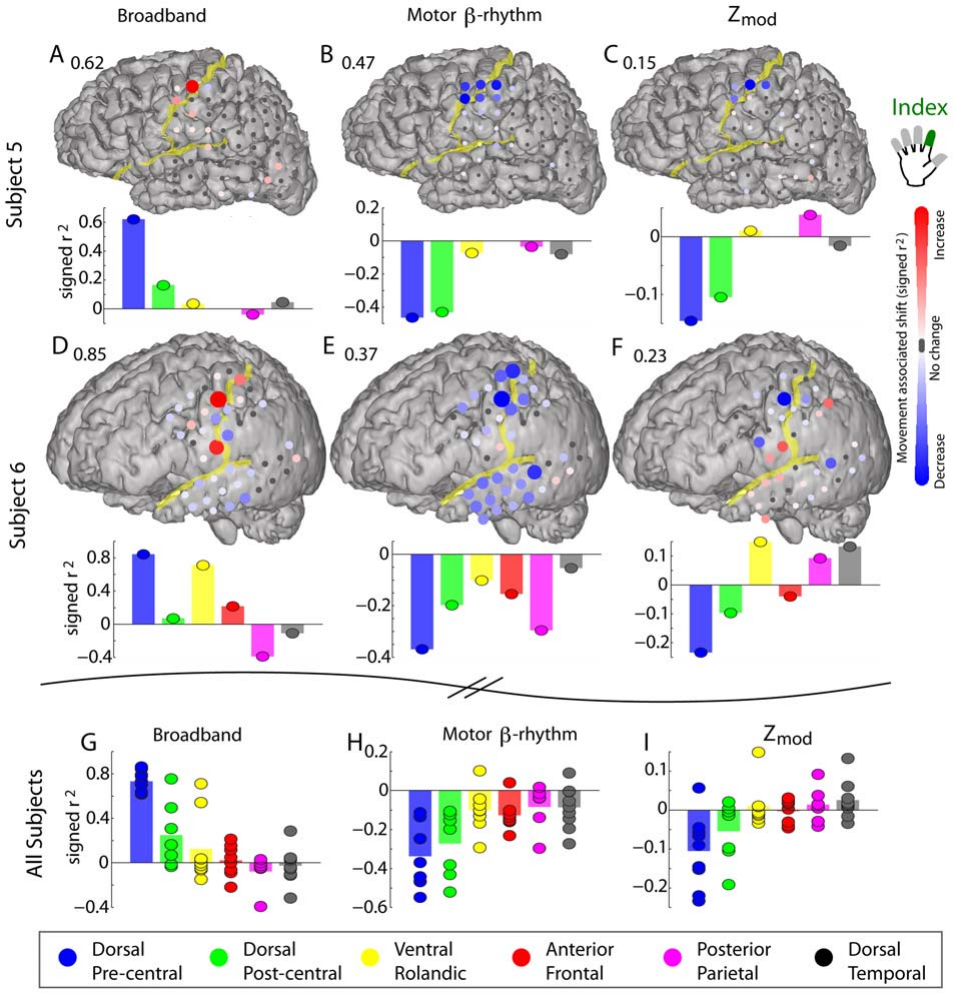
Movement associated shift in rhythmic modulation of local cortical activity for different cortical areas. (**A**) The spatial distribution of shift in broadband magnitude during index movement compared with rest (a signed r^2^ measurement – maximum scaling denoted by the number above the cortical rendering). The bars below denote the maximum signed-r^2^ increase/decrease in each region (Subject 5). (**B**) As in A, but for the shift analytic amplitude of the 12–20 Hz filtered voltage (β-rhythm). (**C**) The shift in the modulation of broadband spectral change with the phase of the 12–20 Hz phase – the signed-r^2^ between the index finger movement epoch and rest epoch projected distributions, as illustrated in Figure 5I&J. (**D–F**) As in A–C, but for Subject 6. (**G**) Maximum signed-r^2^ increase/decrease in broadband spectral change during index finger movement compared with rest, for each region separately for all subjects. Note that the effect is strong and specific to dorsal pre-central gyrus. The bar denotes the average, and each dot denotes a different subject (subjects 1–9). (**H**) As in G, but for the shift analytic amplitude of the 12–20 Hz filtered voltage (β-rhythm). (**I**) As in G, but for the shift in the modulation of broadband spectral change by the phase of the 12–20 Hz rhythm.

Figure 9 shows the movement-related change in rhythmic modulation of broadband cortical activity during index movement relative to rest in different areas. Two subjects are illustrated in A and B, and the results for nine subjects are summarized in G–I. These data again indicate that the greatest change in beta rhythm coupling occurs in the dorsal precentral region.

The phase amplitude coupling palettes in Figures 4, 6, 7, and 8 demonstrate a strong and significant modulation of broadband spectral change with the phase of low frequency rhythms in the δ/θ/β ranges. The variability of the coupling strength across different sites on the lateral cortical surface, and across different behavioral states (movement, rest, visual fixation) is also clear. Often the broadband spectral change is modulated with more than one rhythm at a single electrode site, and multiple couplings are superimposed in the coupling palettes. The predominant phase couplings for different frequency ranges are different.

- β (∼12–20 Hz, “low-β” although precise range varies between sites) most often has a preferred coupling phase of 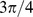 (Figures 6, 7D), and is focused peri-centrally (Figures 6, 7A/E).
- θ (∼4–8 Hz) has a preferred coupling phase of π (Figure 7B) which appears robustly across subjects and across the cortical surface (Figures 6, 7A/C) [33].
- δ (∼1–3 Hz) coupling is seen with different phases in different areas around the lateral cortical surface (Figure 6).

### The conditional relationship between β-amplitude, β-modulation of broadband, and behavior

The structural relationship between modulation of local activity by the β-rhythm and β-amplitude is not simple. In subjects 1–9 (finger movement task), there are 484 electrode sites after rejecting ones with artifacts or epileptic activity. Of these, 297 sites exhibit significant coupling during rest epochs (e.g. “Z_mod_ sites” = 297, p<0.01, uncorrected for multiple comparisons - 4.8 expected by chance). There is a significant decrease in the 12–20 Hz range amplitude in 142 (e.g. “β sites” = 142, p<0.01, uncorrected for multiple comparisons - 4.8 expected by chance). Amongst these 142, 98 (69%) are sites with significant coupling during rest (e.g. “β sites” that are “Z_mod_ sites” = 98). Therefore, these two types of measurement are not spatially correlated in a simple way.

These experiments pose the issue of the basic relationship (if any) between the amplitude of the β-rhythm (12–20 Hz) and the coincident strength of rhythmic modulation. On an epoch-by-epoch basis, β amplitude can be compared parametrically with strength of modulation via a standard Pearson correlation. One hypothesis would be that the degree of rhythmic modulation is determined by a simple relationship with the β-amplitude: with a task-related decrease in β-amplitude, there would be a corresponding decrease in modulation. In order to test this, the epoch-by-epoch correlation was measured between β-amplitude and (projected) modulation strength, performed independently for movement epochs and rest epochs (Illustrated in Figure S17 in Text S1). During rest epochs, there is a clear correlation in many cases. Of the 297 “Z_mod_ sites”, there was significant correlation at 71 sites (p<0.01, uncorrected for multiple comparisons - 3.0 expected by chance; 2 were negatively correlated). Of the 142 “β sites”, 33 showed significant epoch-by-epoch correlation during rest periods (p<0.01, uncorrected for multiple comparisons - 1.4 expected by chance; 3 were negatively correlated). Not surprisingly, 30 of the 33 were at “β sites” that were also “Z_mod_ sites”. Only 3 of the 341 sites that were either “Z_mod_ sites” or “β sites” showed significant correlation during movement epochs (p<0.01, uncorrected for multiple comparisons - 3.4 expected by chance, none were at sites which were correlated for rest epochs).

The conclusions that can be drawn from this are that 1) there is a parametric relationship between β amplitude and broadband modulation by β phase in the resting cortex at many loci, and 2) this relationship is not seen in the active cortex. One interpretation of this is that during movement, these cortical areas are in a lower amplitude regime of β, and that in this lower power regime, the 12–20 Hz amplitude attributed to the rhythmic activity is small compared to the stochastic 1/f portion of the signal, so any parametric relationship disappears (see illustration in Figure S17 in Text S1). This interpretation is supported by the epoch-to-epoch correlation seen during rest at the 30 of 71 sites with modulation during rest that also show movement-associated shift in β amplitude. However, that interpretation does not account for the 41 of 71 sites with a significant correlation, selectively during rest and not movement, but without a significant movement-associated shift in β-amplitude.

### Functional Magnetic Resonance Imaging (fMRI)

**Figure 10:**
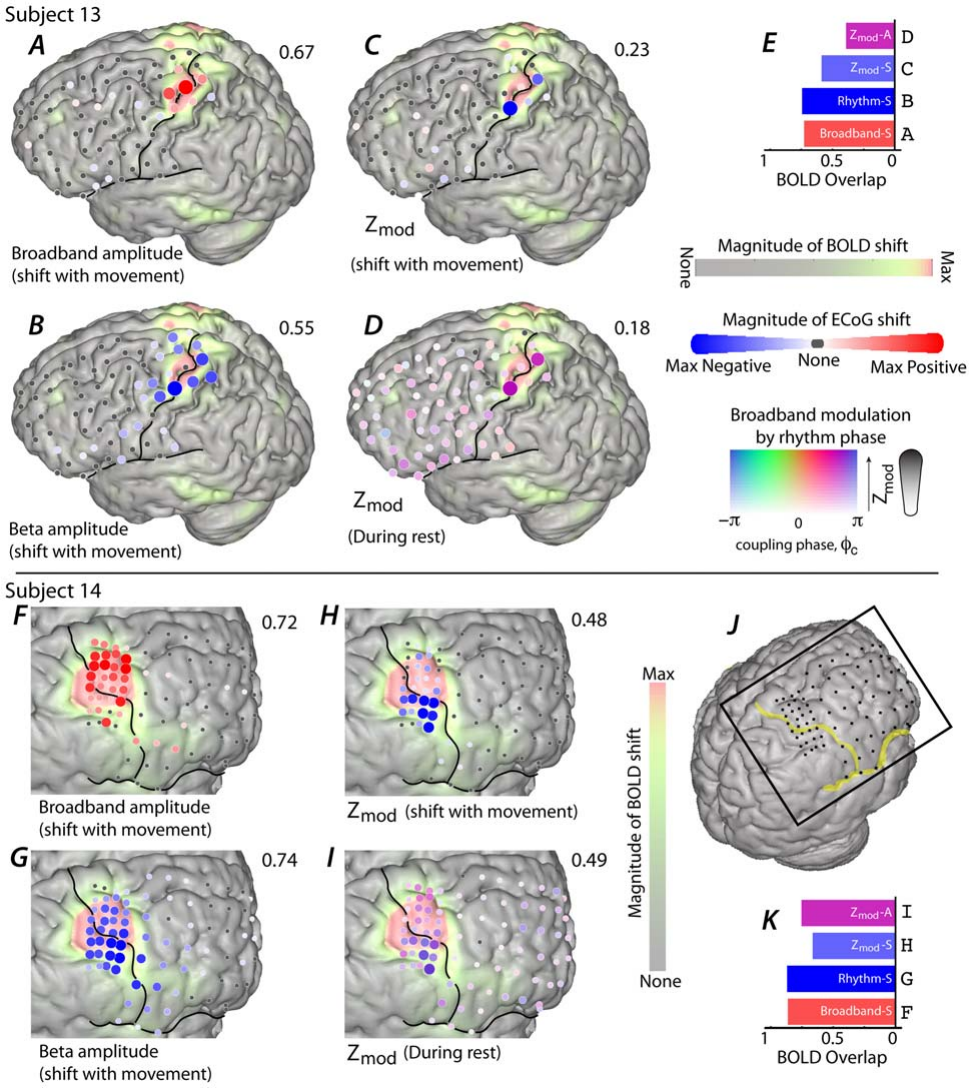
The relationship between fMRI BOLD signal change and ECoG spectral change. (**A**) The magnitude of the fMRI BOLD signal for epochs of thumb movement relative to rest is shown plotted on the brain surface of subject 13. The overlying electrodes show the corresponding shift in ECoG broadband power change (approximated with 65–135 Hz (100 Hz European line noise excluded), with the maximum r^2^ value noted above the cortical rendering. (**B**) As in A, except a comparison of fMRI BOLD shift and 12–20 Hz amplitude shift. (**C**) As in A, except a comparison of fMRI BOLD shift and shift in modulation of broadband by 12–20 Hz phase. (**D**) As in A, except a comparison of fMRI BOLD shift and absolute amount of modulation of broadband by 12–20 Hz phase specifically during rest – the strength of color and diameter denotes the magnitude of modulation, and the color itself denotes what the preferred phase of modulation is. The number above the cortical rendering denotes the maximum Z_mod_ in the array. (**E**) Explicit quantification of spatial overlap from the plots in A–D. (**F–K**) As in A–E, but for subject 14.

In two subjects it was possible to correlate the ECoG recordings with fMRI measures of the BOLD signal, using registration techniques described in the Methods. Finger movement generates a robust BOLD activation, primarily focused in the pre- and post-central gyri, as well as smaller outlying activations (Figure 10). The topography of BOLD changes overlapped the most with the topography of shift in the β rhythm (10B, G). The location of broadband ECoG change with thumb movement also coincided with the largest BOLD changes (10A, F). The least overlap with BOLD occurred with phase-amplitude modulation (10C, H). Topographies of the resting phase-amplitude modulation and the movement-associated change in phase-amplitude modulation had similar overlap with the topography of BOLD change (10D, I).

### Phase coherence

**Figure 11:**
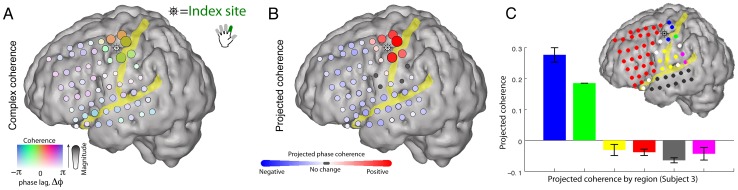
Pair-wise 12–20 Hz phase coherence with a peri-central index-finger specific electrode (subject 3). The index-finger specific site is identified by magnitude of broadband shift, and denoted with a “ship wheel” symbol. (**A**) The pair-wise phase coherence between sites is calculated with the remainder of the array (during rest periods). The magnitude of the phase coherence (max = 0.32) is reflected in the strength of the color and the electrode diameter, whereas the relative phase-lag of the phase coherence is denoted by the color. Note that the spatial pattern of phase coherence clusters by gyral anatomy. (**B**) In order to more clearly isolate spatial changes in phase, the complex phase coherence at each site was projected onto the phase of the site of maximum absolute phase coherence. (**C**) The mean projected phase coherence from each region (shown color coded on inset cortical rendering) is quantified, with error bars denoting the standard error of the mean within each region.

**Figure 12:**
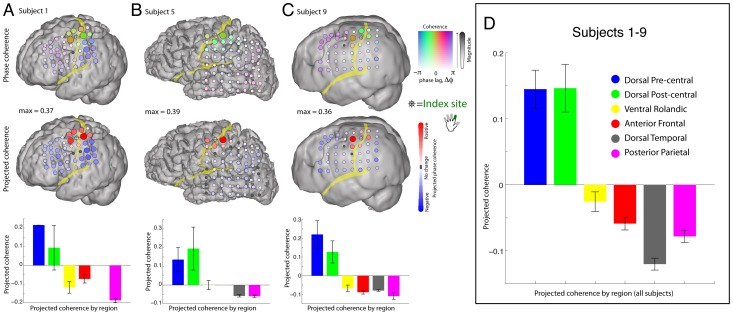
Pair-wise 12–20 Hz phase coherence with a peri-central index-finger specific electrode across subjects. (**A–C**) As in Figure 11, for subjects 1, 5, and 9; maximum phase coherence noted in between cortical renderings. (**D**) Pooled data from subjects 1–9, showing that the 12–20 Hz pair-wise phase coherence is conserved within dorsal pericentral cortex, bounded by the pre-central sulcus anteriorly, and the post-central sulcus posteriorly. Note that the “anti-phase coherence” is most strongly due to introduced phase coherence in the common-average process (π out of phase) in the electrodes that do not otherwise have a large beta rhythm.

To determine the extent to which β-rhythms at neighboring cortical sites are generated by common versus independent sources, we examined the pairwise phase coherence of the β rhythms across sites. In contrast to many studies in which the absolute value of phase coherence (or mean-squared phase coherence) is used, we retain the phase-shift between sites. If there is a strong, coherent rhythm in a large fraction of the electrode array, then the act of re-referencing will introduce false phase coherence, π out of phase. When this occurs, it can be revealed by examining the phase of the phase coherence. Additionally, different motifs in phase coherence, such as those which would be found with propagating waves [25], would be revealed. Figure 11A plots the phase coherence relative to a chosen reference site at which broadband power was maximally correlated with movement of the index finger. The figure shows that phase coherence of the β rhythm is spatially distributed over neighboring sites. Furthermore, by projecting each phase coherence value (i.e. each phase coherence vector in the complex plane) onto the strongest phase coherence value (i.e. a reference vector) we can measure the extent to which phases are aligned among the coherent sites. This projection procedure facilitates the identification of variation in phase across different cortical sites (Figure 11B&C). The spatially coherent rhythms are gyrally delineated along the pre-central and post-central gyri. The phase coherence measure either decreases in magnitude, or “skips” in phase as sulci are crossed. Thus the phase coherence motifs for the motor rhythm with M1 obey sulcal boundaries. As shown in Figures 11, 12, and in Figure S19 in Text S1, this is true across all 9 subjects. The spatial pattern of phase coherence in the 4–8 Hz theta range likewise shows anatomic delineation, but with a different spatial pattern than the 12–20 Hz beta rhythm (see Figure S20 in Text S1).

### Correlations with movements evoked by cortical stimulation

Responses evoked by pair-wise cortical stimulation between adjacent electrodes was systematically documented in four subjects. The sites where stimulation evoked hand movement were all in the pre-central gyrus, and all were coincident with the sparse subset of sites that exhibited significant broadband spectral change for different finger movement types (Figures S7, S8, S9, S10 in Text S1).

## Discussion

### Entrainment of broadband power on phase of underlying rhythm

The major new findings of this study show that cortical population activity, as reflected in broadband power of the ECoG potentials, is significantly modulated with the phase of different rhythmic elements of the cortical surface potential. These relations are comprehensively documented in the “phase-coupling palettes” from various sites on the lateral cortex, which show how the average value of the instantaneous broadband power varies with the phase of a wide range of low frequency rhythms. Notably, local cortical activity, revealed by broadband spectral change, is entrained on the 12–20 Hz beta rhythm in peri-central cortex. When second-to-second changes in entrainment are examined during epochs of movement and rest, there is selective decrease in rhythmic entrainment during movement epochs.

**Figure 13:**
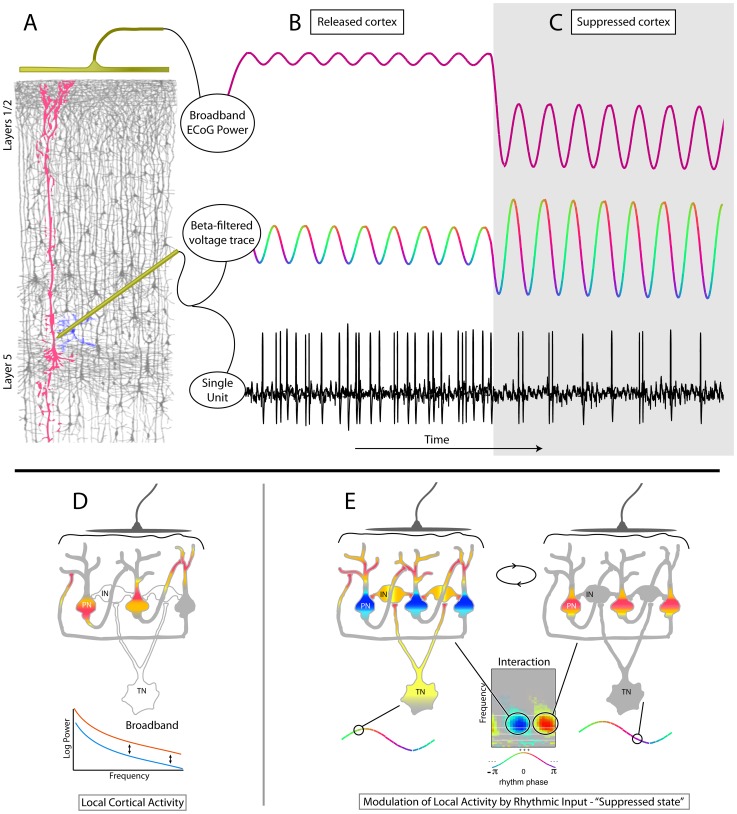
Modes of neural activity with cortical beta rhythm states. (**A**) Modulation of broadband amplitude by underlying rhythm can be thought of as population-averaged spike-field interaction. (**B**) “Released cortex” demonstrates a small amount of broadband power coupling to underlying rhythm phase, and the underlying spiking from pyramidal neurons is high in rate and only weakly coupled to the underlying rhythm phase. (**C**) “Suppressed cortex” demonstrates less broadband power but with higher modulation by the underlying rhythm, while underlying single unit spiking is low in rate but tightly coupled to the rhythm phase. (**D**) A simplified heuristic for how rhythms might influence cortical computation: During active computation, pyramidal neurons (PN) engage in asynchronous activity, where mutual excitation has a sophisticated spatio-temporal pattern. Averaged across the population, the ECoG signal shows broadband increase, with negligible beta. (**E**) During resting state, cortical neurons, via synchronized interneuron (IN) input, are entrained with the beta rhythm, which also involves extracortical circuits symbolized by the input from a synchronizing neuron in the thalamus (TN). The modulation of local activity with rhythms is revealed in the ECoG by significant broadband modulation with the phase of low frequency rhythms. (Note: D–E modified from [30], with permission).

The phase-coupling palettes show particularly strong phase modulation at sites in sensorimotor cortex (Figures 6–8). Consistent with this evidence, neural recordings in monkeys show that action potentials of many motor cortex neurons tend to be entrained at particular phases of local rhythms [23], [24], [25]. In one study about 2/3 of precentral neurons fired about 30 degrees prior to the peak negativity of the local field potential oscillations [20–40 Hz], independently of cortical depth [24]. This is consistent with our finding that broadband ECoG power, which reflects aggregate population spiking activity, peaks prior to the surface negativity phase (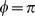) of local beta rhythm (Figures 5, 13).

The features in phase-coupling palettes for dorsal pre- and post-central cortex are clearly more robust than those for other cortical sites, including ventral Rolandic cortex (Figures 6–8). Nevertheless, the palettes averaged over each of these other regions also show similar features during baseline fixation (Figures 6–7). Interestingly the palettes for the lateral brain surface (Figure 7C, 8A) do not show coupling in the gamma range (30–50 Hz), although phase coupling in this range has been observed with ECoG in the occipital cortex [30]. Also, unlike findings in occipital cortex [35], the α (∼8–12 Hz) range most often appears to reflect a transition between discrete motifs in beta coupling and theta coupling, rather than representing a distinct entity in and of itself. Put plainly, we do not see ubiquitous entrainment by an 8–12 Hz “mu” rhythm as might have been anticipated by EEG studies. It is worth noting that it is not necessary to extract a broadband time series in order to see these effects. The coupling motifs can also be seen, albeit less clearly, if broadband spectral change is approximated by band-pass filtered high frequency power as is common in other studies (e.g. with “high gamma” power, illustrated in Figures S3, S4 in Text S1) [33].

The neural mechanism underlying the continuous “diagonal bands” visible in the lower frequencies (δ, θ, α bands) of the phase-coupling palettes may reflect a process in which broadband power is elevated at a fixed time lag relative to the peak of a periodic voltage trace (illustrated in Figure S14 in Text S1). This fixed time lag would then appear as a continuously varying phase lag (diagonal band) across different low-frequency ranges. The periodicity of the voltage determines the base frequency, and the “slopes” of the bands (vertical or angled) are a function of the degree to which both the potential and the broadband are sinusoidal versus a periodic “pulse train”. In other cases, the superposition of multiple rhythms, each of which has a different preferred phase, may give the appearance of a sloped palette (seen clearly in Figure 8 E, G, and J).

### Somatotopy of individual finger movements

The movement-related cortical activity reflected in the extracted broadband is localized to the pre-central gyrus and is spatially quite sparse, showing distinct separate cortical sites for thumb, index, and little fingers in most subjects (Figure 3, Figures S5, S6, S7, S8, S9, S10 in Text S1). Figure 3B/D shows that the overlap between different digits is very small when quantitatively measured. In comparison, single unit studies in the monkey have shown that M1 neurons firing with different fingers are distributed in a largely overlapping fashion [36], [37]. Our findings indicate that these overlapping digit representations nonetheless exhibit a dominant and well-delineated finger representation when cortical activity is summed over the entire neuronal population. Comparable somatotopic organization does not exist for the beta rhythm (12–20 Hz), which is broadly distributed, spanning both the pre- and post-central gyri, and is ubiquitously reduced during movements of any of the fingers. This movement-associated decrease in the β-rhythm exhibits near-complete spatial overlap for different finger movements (Figures 1F, 3, 8O, 9B/E, 10B/G). Moreover, the beta rhythms at neighboring sites are largely coherent (Figures 11&12), which suggests a common mechanism that is inversely modulated related to movements of different fingers.

### The functional mechanisms underlying beta activity

For over a century, cortical rhythms recorded from the brain surface have been known to fluctuate with behavior [38]. However, the physiological origin of these rhythms is poorly understood, and their possible role in brain function remains a topic of debate. The inverse relation between motor activity (including imagery) and the appearance of beta activity in Rolandic cortex has led to the hypothesis that beta, like alpha, is a “resting rhythm”, passively emerging when neural circuits are not actively engaged in computations that desynchronize their activity. In this paradigm, the frequency of this rhythm is determined by the resonant properties of the neural networks that are recruited; these circuits would also include subcortical networks. An alternative and potentially exciting hypothesis proposes that beta rhythms reflect an active mechanism that synchronizes neural populations and suppresses cortical information processing [30]. In such a “suppression through synchronization” hypothesis the input from a “synchronizing locus” in thalamus projects diffusely to an inhibitory population of neurons that, in concert, inhibit pyramidal neurons at their basal dendrites and soma (Figure 13). While our data are potentially consistent with either of these hypotheses, we detail this “suppression through synchronization” hypothesis for further consideration.

### A “suppression through synchronization” hypothesis

What might be the function of these beta rhythms in motor cortex? Perhaps they play a dynamically suppressive role as follows: feed-forward inputs to the large, computationally important pyramidal neurons of motor cortex (Betz cells) are mediated via synaptic inputs to superficial layers, although their somas are in layer 5 [39]. The complex dendritic computations performed as these superficial inputs combine to influence the somatic potential [40], could be interrupted by peri-somatic inhibitory inputs that are rhythmically synchronized across the entire population of Betz cells. Consistent with this idea, selective blockade of inhibitory GABA-A receptors and gap-junctions in layer 5/6 abolishes the beta rhythm [41], and selective GABA agonism with benzodiazepines selectively potentiates the amplitude of the peri-central beta rhythm [42].

At the macroscale of the ECoG, the motor beta-rhythm is spatially synchronized in peri-central cortex (Figures 11&12), where it entrains neural population activity (Figure 7), and transiently releases this entrainment during movement (Figure 9). Such synchronous population activity would prevent the generation of the complex spatio-temporal patterns of activity among pyramidal neurons that are necessary for the execution and coordination of movement. When present, the rhythm keeps the motor cortex in an idling, dynamically “ready”, state – the timings of cortico-cortical distal dendritic inputs are entrained on the population-synchronized GABAeric proximal interneuron input. This scenario is consistent with the fact that broadband measurement of averaged activity from the 10^5^ neurons beneath each electrode is entrained with beta-rhythm of the ECoG (Figure 13). In this way, weak but synchronized interneuron input might keep motor cortical activity in a “dynamically suppressed” state, where neural activity can quickly transition into an active “processing state” when the synchronized input stops.

### The circuit that regulates the synchronization of motor cortex

The inputs of the synchronizing locus to motor cortex likely arise in thalamus as part of a well described cortical to sub-cortical feedback circuit (see illustration in Figure S15 in Text S1). Motor cortex, in addition to its brainstem and spinal projections, also projects to striatum and sub-thalamic nucleus, and these loci follow “direct” and “indirect” pathways to the globus pallidus (pars interna), which, in turn, project to nuclei in the thalamus. The thalamic nuclei close a recurrent feedback loop by sending diffuse input to motor cortex in layer 5 [39], [43]. Supporting this is the observation that the beta rhythm is present at each step of the circuit [8], [9], [43], [44], [45], [46], [47], and physiologic or pharmacologic intervention at the level of the cortex [48], striatum [49], [50], pallidum [51], subthalamic nucleus [52], [53], or thalamus [54], disrupts the beta rhythm throughout the circuit. Furthermore, disruptions in this circuit are correlated with specific motor disease, and interruptions of these loci provide specific therapeutic improvement in the disease with concomitant decrease in beta rhythm [53], [55].

But what generates and drives the beta frequency throughout this circuit? Perhaps the timescale of the beta rhythm reflects the loop time it takes for impulses to travel around the cortical-subcortical circuit, although this is challenged by fact that there are various loop times for different circuits, and also because the resonant frequency of distributed cortical oscillations is not determined by conduction times between synchronized sites [56]. Alternatively, the beta rhythm may be a “cortically local” product of the recurrent network of excitatory and inhibitory neurons within motor cortex. This latter hypothesis is supported by the fact that carbachol applied to rodent cortical slices generates beta-range rhythms in the absence of the rest of the motor circuit [57]. However, there is also evidence against the local-generation hypothesis. When the motor representation of both sides of the body is re-mapped to one hemisphere following perinatal stroke (and the beta rhythm is already present at this stage [58]), the laterality of the beta rhythm is preserved in adulthood, despite the fact that the local representation is not [59].

Finally, an intermediate hypothesis is that the beta oscillation is generated by a pacemaker at a specific subcortical site, but then appears throughout the circuit due to propagation from this site. Using modeling and carbachol injection into the intact rodent, Kopell et al provide evidence that the striatum is the origin of these rhythms [50]. Lesion studies in the cat suggest that the ventral tegmentum generates the beta rhythm [60]. While our study does not directly address the origin of the beta rhythm to distinguish between these hypotheses, our findings do document the functional importance of the beta rhythm as reflected by robust modulation of population-scale broadband activity with beta rhythm phase.

### Bridging scales of measurement

Our ECoG recordings help explain why the beta rhythm is so robust in electroencephalographic recordings from outside the head; namely, it is spatially synchronous across the pre- and post-central gyri, and so this coherent beta rhythm is augmented with respect to background by spatial averaging (Figure 11&12). Furthermore, the different states of the surface rhythm may represent switching between stable modes observed in ongoing surface oscillations. In contrast with the beta rhythm, the broadband spectral change that accompanies movement is asynchronous at the local level [28] and unrelated across cortical regions, so it is washed out in spatial averaging [61] (see heuristic illustration and back-of-the-envelope calculation in Figure S11 in Text S1). The observation that administration of a benzodiazepine GABA agonist produces an increase in magnetoencephalographic peri-central beta range power [42] is potentially consistent with our hypothesis that synchronized thalamic projections to peri-central inhibitory interneuron populations suppress motor cortex. This would suggest that the muscle relaxant effect of benzodiazepines might be mediated by augmenting the synchronizing effect underlying the beta rhythm and suppressing corticospinal output to somatic motor neurons. In functional magnetic resonance imaging (fMRI), the BOLD signal reflects temporally-averaged cortical metabolism of all types (excitatory, inhibitory, glial, etc). A prior study with fMRI and ECoG during simple hand movement showed that the most robust fMRI changes are parametrically associated with 65–95 Hz ECoG power (an approximation of our broadband), specifically in pre-central motor cortex [62]. Outside of this central hand region, a weaker and more spatially diffuse BOLD change was found that correlated with beta-range changes (see Figure 10). Our findings in this study may provide an explanation for this: the pre-central BOLD peak reflects the shift in local cortical activity in the engaged brain area, while the surrounding BOLD change reflects the increased metabolism in adjacent disengaged but also disinhibited cortex.

### The functional importance of the beta rhythm

This study establishes that motor cortical populations are phase-modulated with the beta rhythm, both during long rest periods (minutes of fixation) as well as during brief (1–2 seconds) rest periods within a dynamic finger movement task. During periods of movement, the amplitude of the beta rhythm decreases and this phase-modulation is also diminished. We believe that this reflects a robust mechanism that is fundamental to brain function. Similar experiments in patients with motor disease might reveal how perturbations to this mechanism disrupt cortical function. A “suppression through synchronization” hypothesis explains how diffuse cortical inputs originating from a thalamic locus might allow large regions of the cortex to be functionally suppressed. The expected utility of selectively engaging brain areas results in the dynamic reallocation of metabolic resources, and mediates the coordinated engagement of task-relevant and task irrelevant brain circuits.

## Methods

### Ethics statement

All patients participated in a purely voluntary manner, after providing informed written consent, under protocols approved by the Institutional Review Board of the University of Washington at the Seattle site, and the ethical committee of the Universitair Medisch Centrum Utrecht (in accordance with the Declaration of Helsinki 2004) at the Utrecht site.

### Experimental setting

#### Human subjects

Fourteen patients at two institutions participated in the study (13–45 years old, 8 female, see table in Text S1). Subjects 1–12 were patients at Harborview Hospital in Seattle, WA, USA, and subjects 13–14 were patients at Universitair Medisch Centrum Utrecht in Utrecht, The Netherlands. Each had sub-dural electrocorticographic (ECoG) grids placed for extended clinical monitoring and localization of seizure foci, in the course of the treatment for medically-refractory epilepsy.

#### Electrocorticographic recording

The platinum electrode arrays (Ad-Tech, Racine, WI) were configured as combinations of “grid” (4×8 8×8) and strip arrays, numbering between 32 and 81 in total. The electrode pads had 4 mm diameter (2.3 mm exposed), 1 cm inter-electrode distance, and were embedded in silastic. These arrays were surgically placed on the sub-dural brain surface during the treatment for epilepsy. Subject 14 had a higher density array with smaller electrodes (see Figure 10).

*In Seattle:* ECoG signals were split into two identical sets. One set was fed into the clinical EEG system (XLTEK, Oakville, Ontario, Canada) and the other set was recorded with Synamps2 (Neuroscan, El Paso, TX) biosignal amplifiers at 1 kHz with an instrumental bandpass-filter from 0.3 Hz to 200 Hz. Finger position was recorded in subjects 1–8 using a sensor dataglove (5DT, Irvine, CA).

*In Utrecht:* ECoG data were acquired with a 128 channel recording system (SD-128, Micromed, Treviso, Italy) with 512 Hz sampling rate and instrumental 0.15–134.4 Hz band-pass filter.

ECoG signals were acquired from the experimental amplifiers using the general-purpose BCI2000 software [63], which was also used for stimulus presentation.

#### Functional Magnetic Resonance Imaging measurement (Utrecht only)

Preoperatively, fMRI scans were acquired on a Achieva 3T scanner with a PRESTO [64], [65] sequence (442 scans TR/TE 22.5/33.2 ms, flip angle 10 degrees, FOV = 256×224×160 mm, acquisition voxel size 4 mm isotropic). Functional images were realigned and co-registered using normalized mutual information [66] with an anatomical scan using SPM5 (http://www.fil.ion.ucl.ac.uk/spm/). The anatomical image was segmented in gray and white matter with unified segmentation [67]. Statistical analyses were performed on a single subject basis and therefore no spatial smoothing was applied prior to regression of BOLD against task. A GLM was estimated with one regressor for hand movement activation (a 30 s box car for movement epochs convolved with a standard hemodynamic response function), data were corrected for low frequency drifts by a 128 s high pass filter and corrected for serial correlations with a first order AR model. For each ECoG electrode, the size of the BOLD signal change for movement compared to rest was estimated by the parameter estimates from the GLM (i.e. percentage of signal change with respect to the global mean) that was averaged across gray matter voxels in an 8 mm radius of an electrode. Functional MRI results are displayed as t-maps that were generated by testing the GLM hand movement parameters estimates for statistical significance.

#### Cortical rendering and electrode localization

The relationship between electrode position and gyral anatomy was determined by first rendering the cortical surface from a pre-operative MRI, using either the freesurfer [68] or spm5 [67], [69] environment. Then electrode positions were calculated with respect to the pre-operative MRI from post-operative computed tomography (CT) using the CTMR package of Hermes, et. al., 2010 [70], demonstrated to accurately localize the electrode positions within an error of ∼4 mm (the size of the electrodes). For subject 3, a post-operative CT was not available, so AP and lateral x-rays were used to localize the electrodes on the rendered cortical surface [71].

#### Stimulation mapping

In 4 subjects (2, 7, 8, and 9), electrocortical stimulation mapping (ECS) [72], [73] of motor cortex was performed for clinical purposes. Each such patient underwent stimulation mapping to identify motor and speech cortices as part of his/her clinical care. In this mapping, 5–10 mA square wave current pulses (1 ms in length) were passed through paired electrodes for up to 3 s (less if a response is evoked) to interrupt function, induce sensation, and/or evoke motor responses (see Figures S7, S8, S9, S10 in Text S1 for comparative maps).

#### Fixation task (subjects 1–4, 7–12)

The first type of experiment, fixation, was performed by the subjects fixating with their eyes open on an “X”, on the wall 3 m away, for several minutes.

#### Finger movement task

*Seattle* (subjects 1 to 9): During the finger movement task, subjects were cued with a word displayed on a bedside monitor indicating which finger to move during 2-second movement trials. The subject performed self-paced movements in response to each of these cues, and they typically moved each finger 2–5 times during each trial, but some trials included many more movements. A 2-second rest trial (blank screen) followed each movement trial. There were 30 movement cues for each finger, and trials were interleaved randomly. Finger positions were recorded using a 5 degree-of-freedom dataglove sensor (5 dt, Irvine, CA). Event markers were calculated marking the initiation, peak (denoted 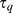), and termination of each movement. This typically yielded 100–150 movements for each finger. “Rest” events (included in 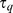) were defined during random periods occurring at least 500 ms from any movement initiation or termination, and separated by at least 250 ms from any other rest event. There were typically 150–250 rest events for each subject. A 37 ms (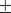
3 ms, SEM) lag occurred between the dataglove position measurement recording and the amplifier measurement.

#### Finger movement task

*Utrecht* (subjects 13–14): During a preoperative fMRI session and during ECoG recordings these patients performed the same hand movement task that consisted of 30 second movement epochs (visually cued thumb/finger flexion at the rate of 2 Hz) alternated with 30 second epochs of rest for 4.5–5.5 min.

### Signal processing

#### Pre-processing

To reduce common artifact, the potential, 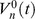, measured at each electrode 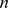 was re-referenced with respect to the common average of all 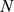 electrodes, 
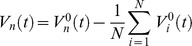
, (channel label, 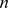, henceforth dropped). Electrodes with significant artifact or epileptiform activity were visually rejected prior to common averaging, and omitted from further analysis.

#### Power spectral snapshots

A set of epochs surrounding events 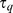 were extracted from 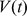; each epoch was of duration 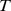 = 1 s, 
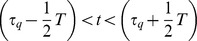
. The epochs were sorted according to cue type 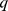, and labeled by their event markers 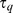. The power spectral density (PSD) of the epoch flanking time 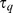 was calculated as
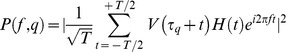
with Hann window [74], 
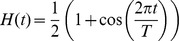
.

#### Dynamic power spectral measures

Time-frequency approximations (dynamic spectra) were made using both a wavelet and a Hilbert transform approach (although these can be equivalent under specific conditions, they are used in a way such that they fill different roles and reflect very different quantities in this study).

The wavelet approach uses a Morlet wavelet [75] of the form: 
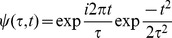
 was convolved with the voltage timeseries to get a time-frequency estimate for every 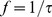:
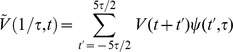
A total of 5 cycles (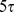) was used to estimate the amplitude and phase of the signal at each frequency for every point in time. In this way a time-varying Fourier component 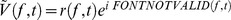, with fixed uncertainty between the confidence in the estimate of the instantaneous amplitude and phase versus the confidence in temporal resolution is obtained at each Hz.

This time-frequency approximation can be used to calculate mean power spectral density as a function of time and frequency in relation to the onset of finger movement from each epoch (Figure 2):
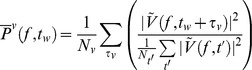
Where 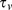 denote onset times of the first movement in response to each cue (total 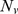), and 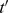 denote inter-stimulus times (total 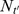). The perimovement time window of interest is denoted 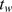; in our case, 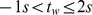.

In the Hilbert transform approach, a complex signal to reflect the timecourse of a functionally-relevant frequency-range band was constructed as follows: The signal 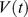 was band-passed using a 3^rd^ order Butterworth filter for a specific range, to obtain the “band-limited” potential, 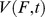, where 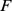 denotes the frequency range (e.g. for “beta”, 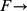
[12–20 Hz], etc.). A complex, analytic, signal, 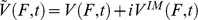
was constructed using the Hilbert transform (e.g. such that the new complex signal satisfies the Cauchy-Riemann conditions for analyticity at all times). This signal may also be expressed in polar notation: 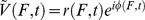. The “analytic amplitude” of the range 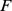 at time 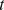 is 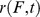 and the “phase” is 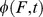. The interpretation of 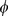 is intuitively difficult, but the most concrete understanding in our context is that the rhythm captured by range 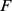 is most surface-positive at 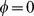, and most surface-negative at 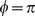 or 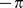 (this interpretation would not be valid for bipolar re-referenced data). Note that 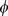 becomes poorly defined as 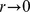.

#### Decoupling the cortical spectrum to separate rhythmic activity away from broadband change

The decoupling process is described and illustrated in full detail in the main text and supplement to the Miller, et. al. 2009 manuscript [31].

In a principal component decomposition of spectral change, the samples of the PSD, 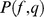, (total 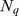), were first normalized prior to decomposition.
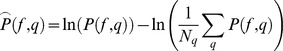
A singular value decomposition is used to determine the eigenvalues 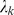 and eigenvectors 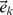 of the correlation matrix 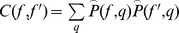.

These eigenvectors, 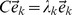, the “Principal Spectral Components” (PSCs), reveal which frequencies vary in power together, and are ordered by magnitude of corresponding eigenvalue: 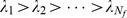
(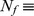
number of frequencies and also eigenspectra). If we define the rotation matrix 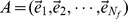, then the projection, 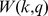, of each individual original spectrum in the ensemble onto the new basis vector 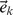 is 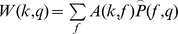.

The inverse rotation matrix 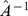, 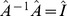, allows us to compare and visualize specific subsets of PSC components with the original full spectrum in frequency space. The 2^nd^ to 4^th^ PSCs typically capture rhythmic power spectral phenomena, and power spectra can be reconstructed with and without this rhythmic influence: 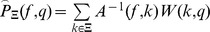.

If the 2^nd^ to 4^th^ PSCs are omitted, 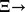
[1,5…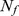], then PSDs can be reconstructed where changes in rhythmic spectral phenomena are mostly removed (although there may be residual variance in the decomposition, or some rhythmic influence in all cases – see Figure 2). If 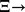
[2–4], then PSDs can be reconstructed where changes in rhythmic spectral phenomena are mostly isolated.

Then, the time-dependent, normalized, dynamic spectrum, 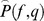, can be obtained in parallel fashion to the spectral snapshots.
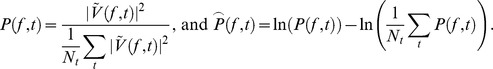
The reflection of the 1^st^ PSC (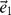) in the dynamic spectrum can be estimated by projecting the dynamic spectrum onto it: 
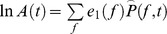
. We call it 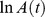 here, because it approximates the logarithm of the timecourse of the coefficient of a power law in the cortical spectrum of the form 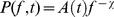 [28]; it is smoothed with a Gaussian window of 50 ms standard deviation, z-scored, and exponentiated to obtain the “broadband” traces plotted in Figures 1D, 2D&N, 3C, 4C, 5E, and Figures S5, S6, S7, S8, S9, S10 in Text S1 (e.g. time varying estimates of the coefficient of the power law spectrum). The broadband power timecourses are robust estimates of behaviorally relevant local cortical firing rate [26], [27], [31], [76]. Because the quantity 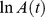 is approximately normal-distributed (see Figure 4F), we express it in Z-score units, and, for notational brevity, denote it 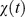 in connection to the broadband power law it reflects. Note that the smoothing and re-exponentiation apply only to the broadband illustrations (i.e. insets in pink in Figures), and not to calculation of coupling to rhythmic phenomena, nor for any other analysis.

#### Broadband coupling to low-frequency phase (illustrated in Figure 4)

The coupling between rhythm phase and local cortical activity was estimated by calculating the average log-broadband amplitude 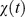 as a function of the rhythm phase 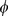 in small phase intervals, 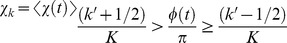, where 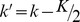 (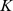 total intervals). For example, for K = 24 (the number used in these analyses) and k = 13, then 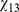 represents the mean log-broadband when the phase of the low frequency rhythm is in the interval between 0 and 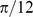. The center of each interval is denoted 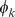, so 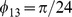. To get a full picture of the strength and preferred phase of coupling across a range of frequencies, the wavelet-obtained rhythms at each frequency are used to build up a “palette” of 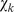 at each frequency. This has an advantage in that ranges of coupling motifs and also distinct coupling to different rhythms are revealed, in many cases, as separate phenomena. This separation is revealed because there are different preferred phases of coupling.

#### The coupling vector and trial-by-trial statistics (illustrated in Figure 5)

To condense the range of frequencies composing a given rhythm into one measure, the Hilbert transform was applied (as described above), with frequency range chosen based upon inspection of the palette (see Figure S2 in Text S1). This allows for the calculation of a “coupling vector” by taking the dot product 
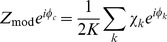
. Z_mod_ is the tmagnitude of coupling between phase of the rhythm and the log-broadband amplitude (because we z-score 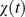, Z_mod_ is roughly the amount of variation in the z-score that correlates with the phase of the rhythm concerned), and 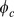 is the preferred phase of this interaction. This can be calculated on a trial-by-trial basis, breaking up the data into smaller epochs of continuous movement or reset and calculating a coupling vector for each epoch. To assess the distribution of coupling for 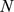 trials of a given type, one cannot simply compare the contribution of trial 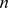 to the distribution of coupling values as 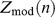 because if 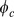 is not reproducible from trial-to-trial, then 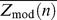 can have a large value even on trials in which the preferred coupling phase is opposite to that of the majority of other trials in the distribution. In other words, the fact that 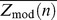 must be nonnegative would strongly bias the distribution of 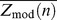 values so that the mean of the distribution can be significantly greater than zero even when there would be no underlying coupling of consistent phase. For this reason, the projected distribution of values 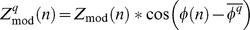 is used, where 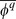 is the preferred phase of the mean coupling vector for trials of type 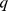: 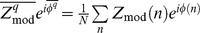. The quantities 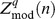 can be negative or positive and can therefore have a distribution significantly overlapping with zero (indicating an absence of reliable phase modulation). For each type of trial, the distribution of coupling can be assessed using the distribution 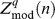. We demonstrate the significance of these measures using error bars that represent 3 times the standard error of the mean. This method has an advantage over the “large-time shift” bootstrapping approach [34], [77] because the data may be segregated into smaller time trials, and statistics may be computed by comparing the distribution of values for trials of one behavioral state versus trials of a different behavioral state. It may also be used to compare discontinuous trials of one type (or all trials concatenated) versus 0. This allows us to examine significant shifts in phase amplitude coupling during different trial types (where 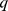 = “rest”, vs 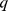 = “thumb movement”, etc.) and also to assess the significance of each independently versus zero. The preferred phase of this coupling vector, 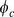, can be thought of as the point where, when averaged across the population of neurons beneath each electrode, action potentials are most likely to occur and the magnitude of the vector Z_mod_ is the mean firing rate correlate of spike-field coupling (Figure 13).

#### Phase coherence of rhythms (Figures 11&12, Figures S18–20 in Text S1)

The pair-wise phase coherence relative to a reference “seed” site was examined. The complex portion was retained because, in the setting of a dominant, distributed, rhythm, the choice of common average reference introduces phase coherence with brain regions where the rhythm is otherwise absent. However, introduced phase coherence will be approximately π radians out of phase with the dominant rhythm. The method to measure phase coherence employed in this paper reflects only a part of the full phase coherence, but is sufficient to address the spatial distribution of pairwise phase coherence. For the complex rhythm 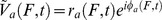 (“seed” electrode 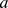, frequency range 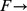
[12–20 Hz]), the complex phase coherence [78] with another electrode 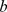, for epoch 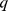 (during time 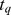) is: 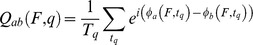. In this manuscript, the “seed” electrode is the motor electrode with the strongest index finger movement-associated broadband change (method illustrated in Figure S18 in Text S1). The complex phase coherence used throughout the manuscript is the average of the complex phase coherences from all rest epochs (e.g. 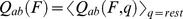); coherence during movement is deferred to other study. In order to illustrate the boundaries and dominant motifs in this phase coherence more clearly, the complex phase coherences for all electrodes in an array are projected onto a unit vector in the direction of the phase lag of the site that has the highest absolute phase coherence: if we denote the pair-wise complex phase coherence 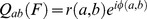, and the pair with maximum phase coherence (e.g. highest 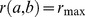) as 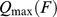 with phase shift 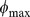, then the projected phase coherence is 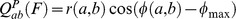.

#### Quantifying overlap in spatial distribution

In order to quantify the overlap between spatial extent and degree of change in two different measures, we use a resampling metric. For measures of types 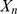 and 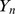 (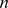 denoting electrode), the simple dot product between the two gives a “true” overlap, 
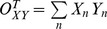
, that can be compared to surrogate distributions for quantification of magnitude and significance. The spatial overlap metric is the true overlap divided by the maximum possible overlap: 
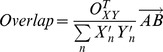
, where 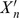 denotes the distribution 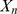, re-sorted in ascending order. To estimate the significance of this overlap using resampling, the electrode labels are then randomly scrambled (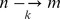), and a surrogate overlap is obtained 
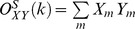
. This is done 10^6^ times. A p-value for the significance of the overlap is the percentage of 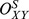 that are greater than 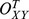 (or the percentage that are less in the case that 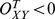).

### Some methodological considerations

- In some subjects (#2,5,6) spatial somatotopy of finger movements could not be adequately documented because these had only partial coverage of the precentral gyrus.
- The findings in this manuscript do not represent an independent verification of the somatotopic phenomenon reported in [31] because some of the recordings were from the same subjects.
- Cortical rendering quality was intrinsically tied to the quality of clinical MRI. Because clinical scans often had very poor slice thickness, reconstructions in some cases were quite coarse. However, they were all sufficient to identify sulcal boundaries and appropriately segregate electrode position by anatomy.
- The analysis metrics used in this paper to measure the influence of rhythms on local cortical activity in ECoG were chosen because they are felt to be intuitive and they integrate with our larger body of work. However, as demonstrated in the Figures S3, S4 in Text S1, the finding of coupling of the motor rhythm to local cortical activity would also be revealed by methods that others have used [33], [34], [35].
- There are likely multiple β-rhythms, and many have distinguished between a “β1” and “β2”. The 12–20 Hz β-rhythm range empirically chosen for this study based upon palettes from pre-central electrodes with robust movement-associated broadband changes (see Figure S2 in Text S1) lies in the low end of the classic 12–30 Hz β-range. Also, it is close in frequency range to the classically described “μ” rhythm found in peri-central EEG.
- Spectral elements that contribute to tracking the lower frequency rhythm phase will be different for the rhythm at each frequency – so, for a 30 Hz rhythm, only the spectral elements above ∼60 Hz can reasonably track the phase at 30 Hz.
- During movement, the β-rhythm amplitude decreases in peri-central cortex, as does the magnitude of the modulation. This might lead the reader to the conclusion that the two are completely correlated, but this is not the case. This is most easily illustrated empirically – when the spatial distribution of the two is examined side-by-side (as is done in Figures 8,9,10), they plainly have different patterns of change with movement, and the shift in the *influence of the β-rhythm* is clearly different than the shift in the *amplitude of the β-rhythm*.
- The choice of common average reference (CAR) was used because of the generally large size of the electrode arrays, and it provides a generic approach. One could, however, have regressed out the mean (to account for varying contact impedances), used pair-wise re-referencing (which would diminish the measurement of phase-synchronized rhythms), or used a single electrode as reference (which would individually confound every channel, but in a predictable way). The conclusions of this paper are robust against such choices. The consequence for CAR in phase coherence estimate was addressed by keeping the complex value of the phase coherence, projecting all phase coherence measures onto the phase lag at the site of greatest phase coherence, and examining the resulting cortical plots for spatial clustering.
- The method does not “self-consistently” produce modulation. When applied to a random-walk timeseries, no modulation is observed (see Figure S13 in Text S1). When applied to synthetic data where broadband signal is embedded modulated by the phase of a coincident rhythm, results similar to our measurements emerge (see Figure S12 in Text S1). As Kramer et. al. note [79], sharp edges or severely asymmetric waveform can produce self consistent modulation; they propose four criteria in their manuscript to assess for this, and our data satisfy all four criteria.
- Broadband spectral changes and β-rhythm changes happen both in of the presence or absence of a movement-onset event related potential (ERP) [9].
- We are spatially under-sampling. For every 1 cm^2^ of cortex, there is only ∼0.04 cm^2^ of platinum exposed to the cortical surface, and, within this area, there are known to be multiple functionally-specific sub-domains [80].
- Although shifts in preferred phase of β-phase to broadband modulation are important and possibly significant, we choose to defer them to other studies. Any statistical measure comparing them will likely have built in assumptions - e.g. to generate a distribution of phases from each condition to compare, one will have to weight each phase measure by the degree of importance.
- One interesting question is: what is the relationship, spatially, between the frequencies that modulate broadband activity in an area, and the power of the signal in that frequency range? Might there be (as one reviewer posed, and with which we agree) a difference between the spatial distribution in the amplitude of a rhythm and the regional sensitivity of local cortical activity to the influence of rhythmic input? In other words, is broadband modulation by a rhythm (Z_mod_) in a given area simply explained by the amplitude of that rhythm? The partial spatial overlap between the changes in rhythm amplitude and the changes in phase-modulation data suggest that such an effect may be present in a subset of the electrodes we measured, but is not the general rule. In practice, this is a technically difficult question to resolve, because the brain rhythms are superimposed on a 1/f background and the amplitude of a band-pass filtered frequency range reflects this superposition. In principle, one could fit the 1/f background at high frequencies, subtract it, and then correlate the residual “rhythm amplitude” with Z_mod_ at that site. The correlation across electrodes would then potentially address this question. However, the fact that the estimate of phase of a rhythm can be affected by the relative ratio of “residual rhythm amplitude” to superimposed 1/f power within a frequency range, sets up a potentially circular and self-consistent set of analyses. Our present study instead compares the spatial distribution of shift in rhythm amplitude to Z_mod_, where no simple spatial relationship is found. We defer direct comparison of extracted rhythm amplitude to Z_mod_ to future studies which would be explicitly constructed to address this circular issue, as this would involve analysis techniques that are significantly more esoteric than those employed here. For example, one such approach might be to extract rhythm amplitude using recursive subtraction of an oscillation with assumptions about phase stability - one would first have to rigorously show that the rhythm was sinusoidal in shape.

## Supporting Information

Text S1 The supplement, Text S1, contains a variety of material that is designed to provide further insight for the more curious reader, further methodology for the more technical reader, and further evidence for the more skeptical reader. It illustrates the interaction between broadband amplitude and rhythm phase using multiple alternate and independent methods of calculation (fures S1, S3, S4). The averaged coupling palette from finger-specific electrodes across many subjects shows that the most robust entrainment in pre-central motor cortex is between 12–20 Hz (Figure S2). The somatotopic representation of individual fingers, and illustrative timecourses (as in Figure 3) are shown in detail for 6 additional subjects (Figures S5, S6, S7, S8, S9, S10). The close correlation between somatotopic representation in broadband and electrocortical stimulation (ECS) is shown for the patients where ECS was performed (Figures S7, S8, S9, S10). We show simple schematics for the relationship between EEG and ECoG for the synchronous (rhythmic and ERP) and asynchronous aspects of the cortical surface potential; a back-of-the-envelope calculation is used to measure the relative contributions of these phenomena (Figure S11). We implement simple heuristics to generate simulated timeseries which have phase entrainment of a 1/f - type signal on an imposed oscillation, illustrating how our analysis techniques extract these phenomena in the expected way (Figure S12). We then apply our analysis techniques to the negative control case of simple 1/f-type colored noise to show that the methods do not self-consistently report phase entrainment (Figure S13). A simple heuristic illustration shows how, and why, the “diagonal bands” in some coupling palettes might exist, in the case where broadband power is elevated at a fixed time lag relative to the peak of a periodic voltage trace (Figure S14). We then illustrate a candidate simple large-scale mechanism for the generation of rhythmic phase-entrainment on local cortical activity (Figure S15). For those readers interested in the preferred phase of entrainment as a function of anatomic region, this is explicitly quantified in Figure S16. The conditional parametric relationship between broadband amplitude, beta (β, 12–20 Hz) amplitude, and magnitude of phase entrainment is further explored and illustrated in Figure S17. Finally, the supplemental material highlights and illustrates phase coherence in further detail, beginning with a simplified illustration of pair-wise phase coherence relative to a reference “seed” site, in Figure S18. Adding to Figures 11 and 12, the pair-wise 12–20 Hz coherence between all sites, and a peri-central index-finger specific site, is shown for the remainder of the subjects, in Figure S19. Figure S20 contrasts the different (yet both gyrally constrained) spatial distributions of coherence for the theta (4–8 Hz) and beta (12–20 Hz) frequency ranges.
(PDF)
