# Supplementary material for: Human Motor Cortical Activity Is Selectively Phase-Entrained on Underlying Rhythms
Source: PLoS Comput Biol. 2012 Sep 6;8(9):e1002655. doi: 10.1371/journal.pcbi.1002655 (PMC3435268; doi:10.1371/journal.pcbi.1002655)
Supplement: Text S1 — The supplement, Text S1, contains a variety of material that is designed to provide further insight for the more curious reader, further methodology for the more technical reader, and further evidence for the more skeptical reader. It illustrates the interaction between broadband amplitude and rhythm phase using multiple alternate and independent methods of calculation (fures S1, S3, S4). The averaged coupling palette from finger-specific electrodes across many subjects shows that the most robust entrainment in pre-central motor cortex is between 12–20 Hz (Figure S2). The somatotopic representation of individual fingers, and illustrative timecourses (as in Figure 3) are shown in detail for 6 additional subjects (Figures S5, S6, S7, S8, S9, S10). The close correlation between somatotopic representation in broadband and electrocortical stimulation (ECS) is shown for the patients where ECS was performed (Figures S7, S8, S9, S10). We show simple schematics for the relationship between EEG and ECoG for the synchronous (rhythmic and ERP) and asynchronous aspects of the cortical surface potential; a back-of-the-envelope calculation is used to measure the relative contributions of these phenomena (Figure S11). We implement simple heuristics to generate simulated timeseries which have phase entrainment of a 1/f - type signal on an imposed oscillation, illustrating how our analysis techniques extract these phenomena in the expected way (Figure S12). We then apply our analysis techniques to the negative control case of simple 1/f-type colored noise to show that the methods do not self-consistently report phase entrainment (Figure S13). A simple heuristic illustration shows how, and why, the “diagonal bands” in some coupling palettes might exist, in the case where broadband power is elevated at a fixed time lag relative to the peak of a periodic voltage trace (Figure S14). We then illustrate a candidate simple large-scale mechanism for the generation of rhythmic phase-entrainme [file pcbi.1002655.s001.pdf]

Text S1, Supplemental material for the manuscript:

"Human motor cortical activity is selectively phase-entrained on underlying rhythms",

by KJ Miller, D Hermes, C J Honey, AO Hebb, NF Ramsey, RT Knight, JG Ojemann, EE Fetz

List of supplemental figures:

1) Alternate illustration of rhythm phase to broadband amplitude comparison.
2) Phase-Broadband coupling in pre-central motor cortex, strongest between 12-20Hz
3) The phase coupling palette compared with the "Cross-frequency Coupling Measure".
4) Coupling to a filtered high frequency range.
5) Electrocorticographic finger somatotopy, subject 1
6) Electrocorticographic finger somatotopy, subject 4
7) Electrocorticographic finger somatotopy, with stimulation positive sites, subject 2
8) Electrocorticographic finger somatotopy, with stimulation positive sites, subject 7
9) Electrocorticographic finger somatotopy, with stimulation positive sites, subject 8
10) Electrocorticographic finger somatotopy, with stimulation positive sites, subject 9
11) The reflection of synchronous and asynchronous (broadband) phenomena in EEG and ECoG.
12) Generation of a simulated timeseries and validation of signal processing technique
13) Application of signal processing technique to colored noise.
14) A possible explanation for the diagonal bands in some coupling palettes
15) A candidate large-scale mechanism for the generation of this rhythmic influence on local cortical activity
16) Preferred phase of modulation by anatomic region, pooled across all subjects.
17) The conditional relationship between broadband, beta (β, 12-20Hz), and modulation.
18) Illustration of pair-wise phase coherence relative to a reference "seed" site.
19) Pair-wise 12-20Hz coherence with a peri-central index-finger specific electrode across subjects.
20) A comparison of coherence with an index finger "seed site" for the theta (4-8Hz) and beta (12-20Hz) ranges.

Supplemental table: Patient characteristics

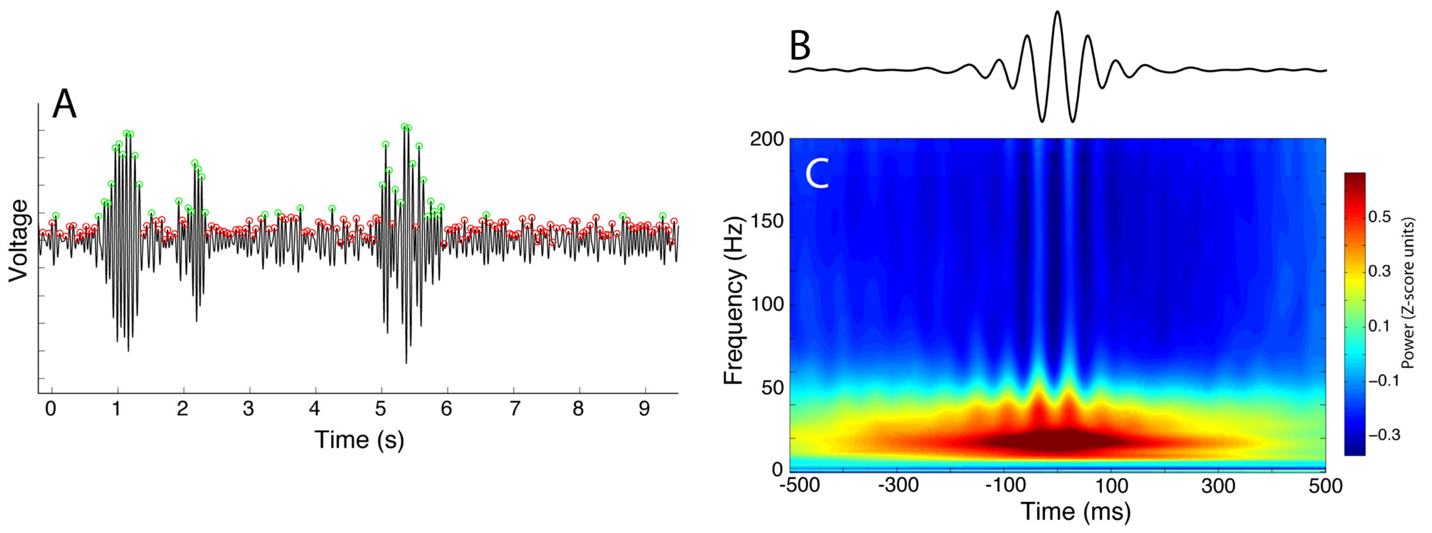

Figure S1: **Alternate illustration of rhythm phase to broadband amplitude comparison, from a pre-central electrode in subject 5. (A)** 10 minutes of common-average re-referenced potential from a motor-area electrode is filtered for the beta range 12-20Hz using a 3rd order Butterworth filter, and the time of top 25% in power peaks are denoted (shown in green circles, bottom 75% in red). **(B)** The average of the raw potential, centered at times of top 25% of peaks (green from A) is shown – The beta rhythm clearly falls out of this. **(C)** The mean spectrogram (globally z-scored), averaged over times of top 25% of peaks (green from A) is shown. It reveals that ***1:*** Coupling between the broadband (vertical lines that extend to the top of the spectrum) and the beta rhythm. ***2:*** The higher power of beta is associated with lower power in broadband (i.e. the spectrogram is yellow-red in the beta range, and blue at higher frequencies).

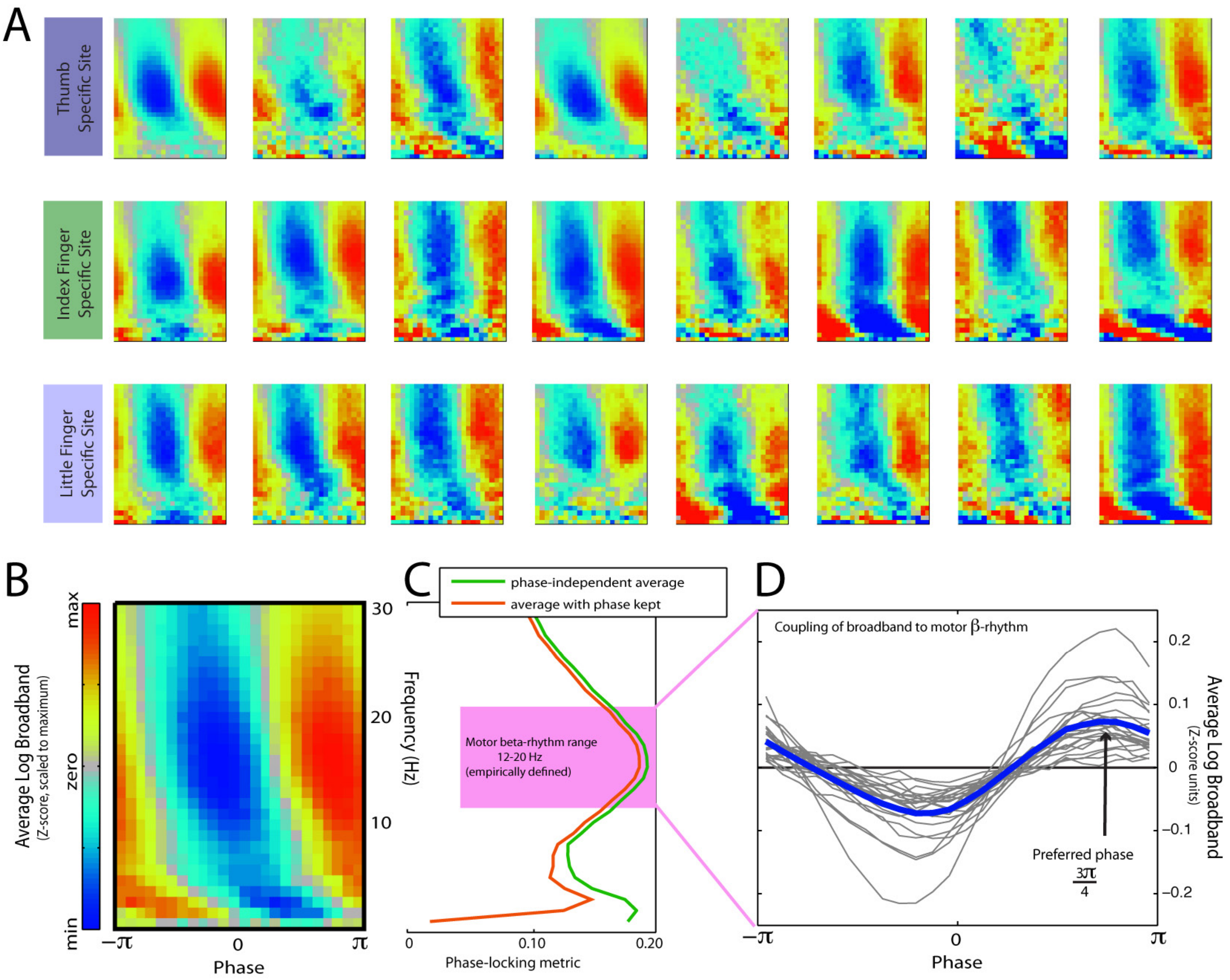

Figure S2: **Phase-Broadband coupling in pre-central motor cortex, strongest between 12-20Hz**. In **(A)**, the phase-broadband coupling palette is shown for 3 motor cortical electrodes (each one is the most specific electrode for the movement of thumb, index, or little finger) for subjects 1-4, and 6-9 during the finger movement task. Coupling to the beta range can be seen in every case, palettes from 1-30Hz only. In **(B)**, the average palette from all of the electrodes in **(A)** is shown. The "phase-locking" metric (dot product of each row in the palette in **(B)** with a sinusoid) is shown in **(C)**, allowing an empiric definition for the beta range (12-20 Hz). In **(D)**, the average of the broadband, sorted by phase of the beta rhythm for all 24 sites in **(A)** are shown in gray (the average of these gray traces is shown in blue). Note that local activity (broadband) is peaked at the falling phase of the beta rhythm (i.e., the preferred phase is 3π/4) in each case.

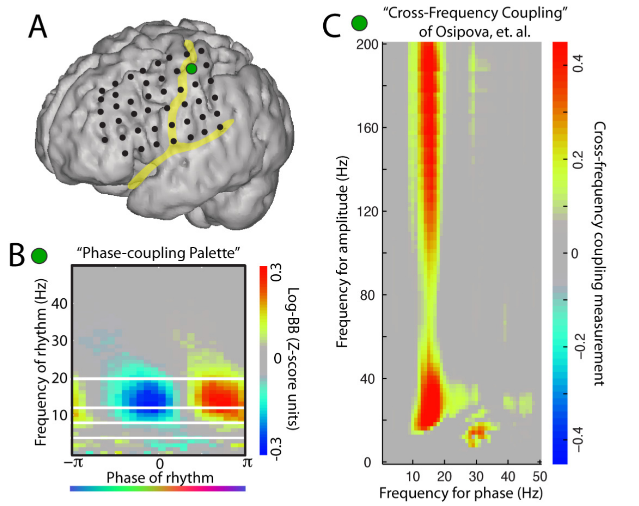

Figure S3: **The phase coupling palette compared with the "Cross-frequency Coupling Measure". (A)** Voltage recorded from peri-central electrode site (green dot) in subject 1. **(B)** Phase-coupling palette calculated using the method from this manuscript. **(C)** Similar findings are obtained using the "Cross-frequency coupling" method employed in the Osipova et al manuscript [1].

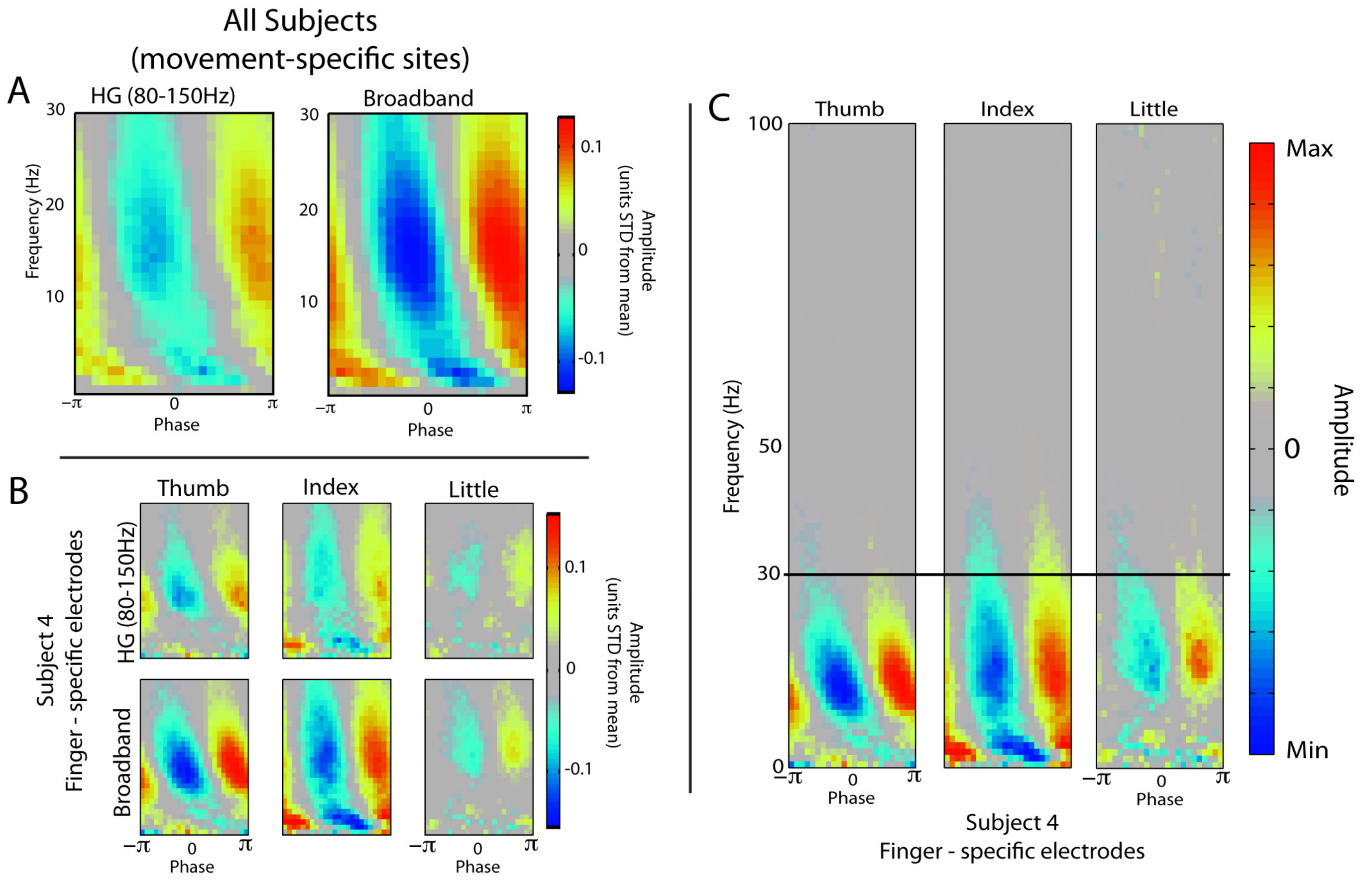

Figure S4: **Coupling to a filtered high frequency range.** The coupling is present if the "high-gamma" (80-150Hz) range of the Canolty et al manuscript [2] is used to measure coupling instead, but the effect is weaker (note that the frequencies in A-B only go up to 30Hz. **(A)** Average of 3 electrodes most significant for thumb, index, and little finger movement, during finger movement task from subjects 1-4, 5-8 during the finger movement task. **(B)** In individual electrodes for subject 4, shown with frequencies from 1-30Hz. **(C)** Subject 4, with coupling up to 100Hz to show that the effect is not present above ~35-40Hz (plots individually scaled to maximum, unlike in A and B).

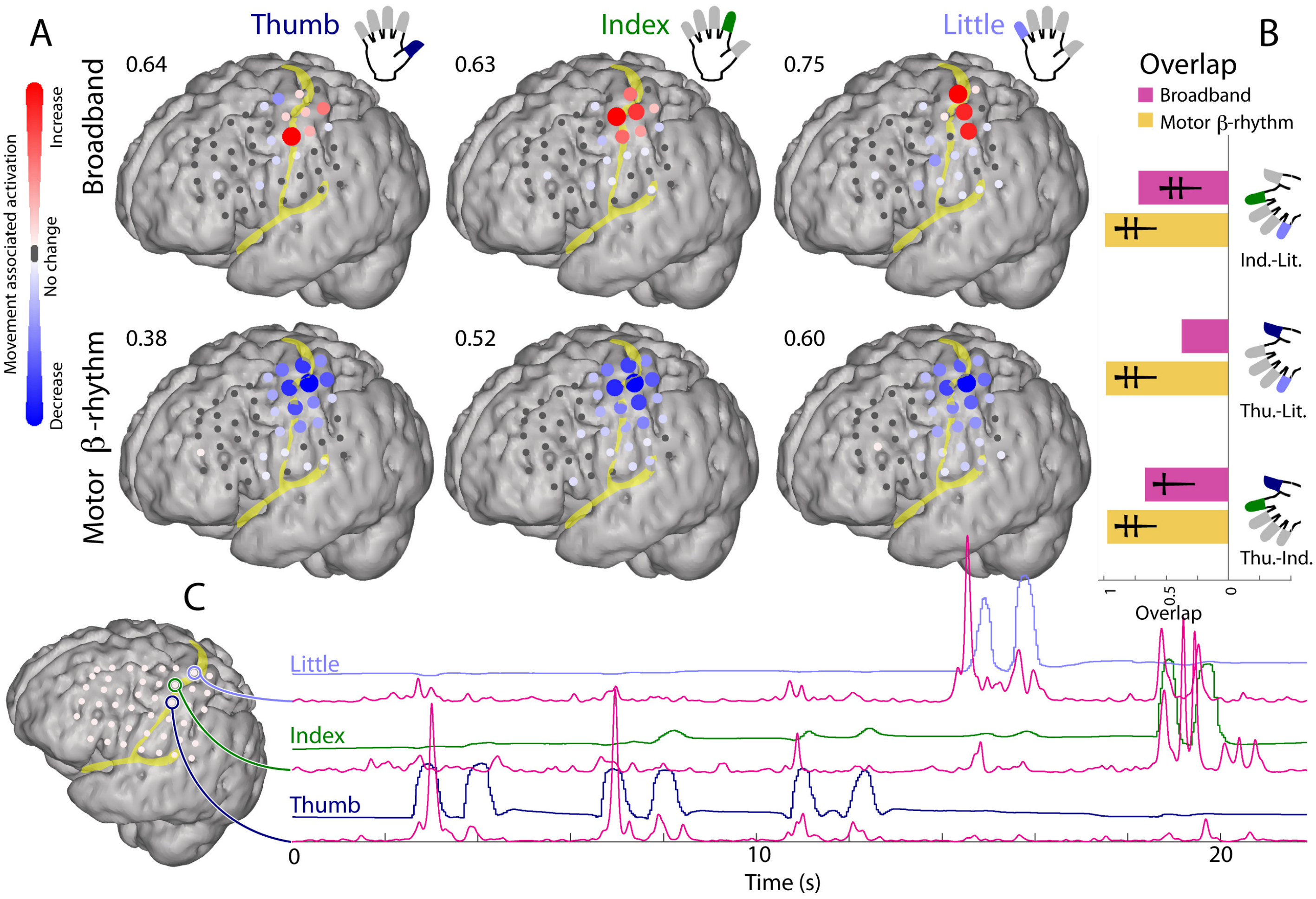

Figure S5: **Electrocorticographic finger somatotopy, subject 1 (as in Fig 3 of the main text)**. **(A)** The spatial distribution of change during thumb, index, and little finger movement-associated change (a signed r^2 measurement) in broadband spectral change (spatially focal, non-overlapping between different fingers), and the 12-20Hz motor rhythm (spatially broad, strongly and significantly overlapping between fingers). Subject 3 illustrated. **(B)** Numerical quantification of spatial overlap between finger-movement associated change. Resampling significance, single cross denotes p<0.01, double denotes p<0.001, that the overlap happened by chance. **(C)** Traces of thumb (dark blue), index (green), and little finger (light blue) position, along with the timecourse of broadband spectral change from 3 electrode sites. Note that each broadband trace correlates with the position of only one of the fingers and not the others.

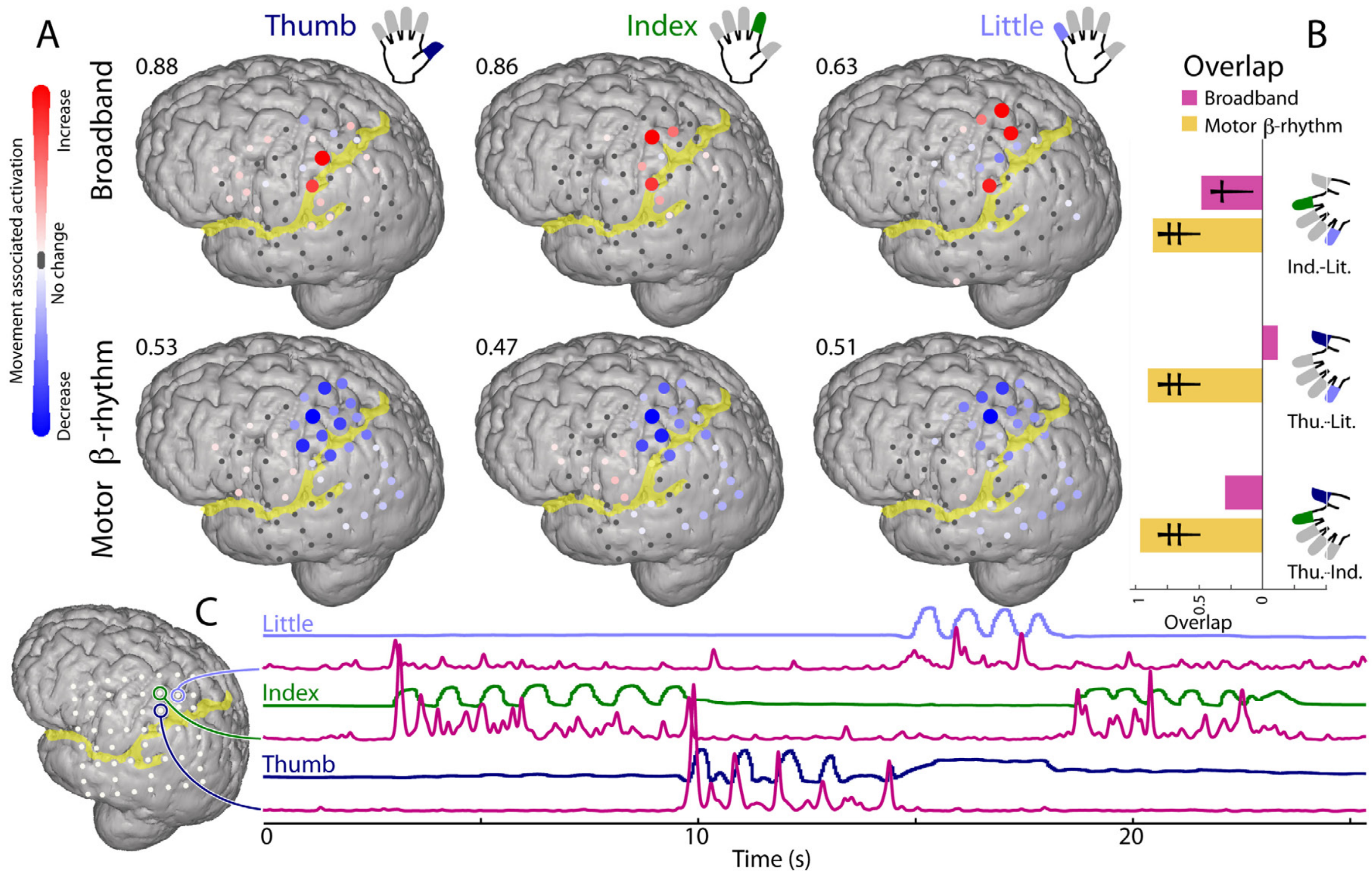

Figure S6: **Electrocorticographic finger somatotopy, subject 4 (as in Fig 3 of the main text)**

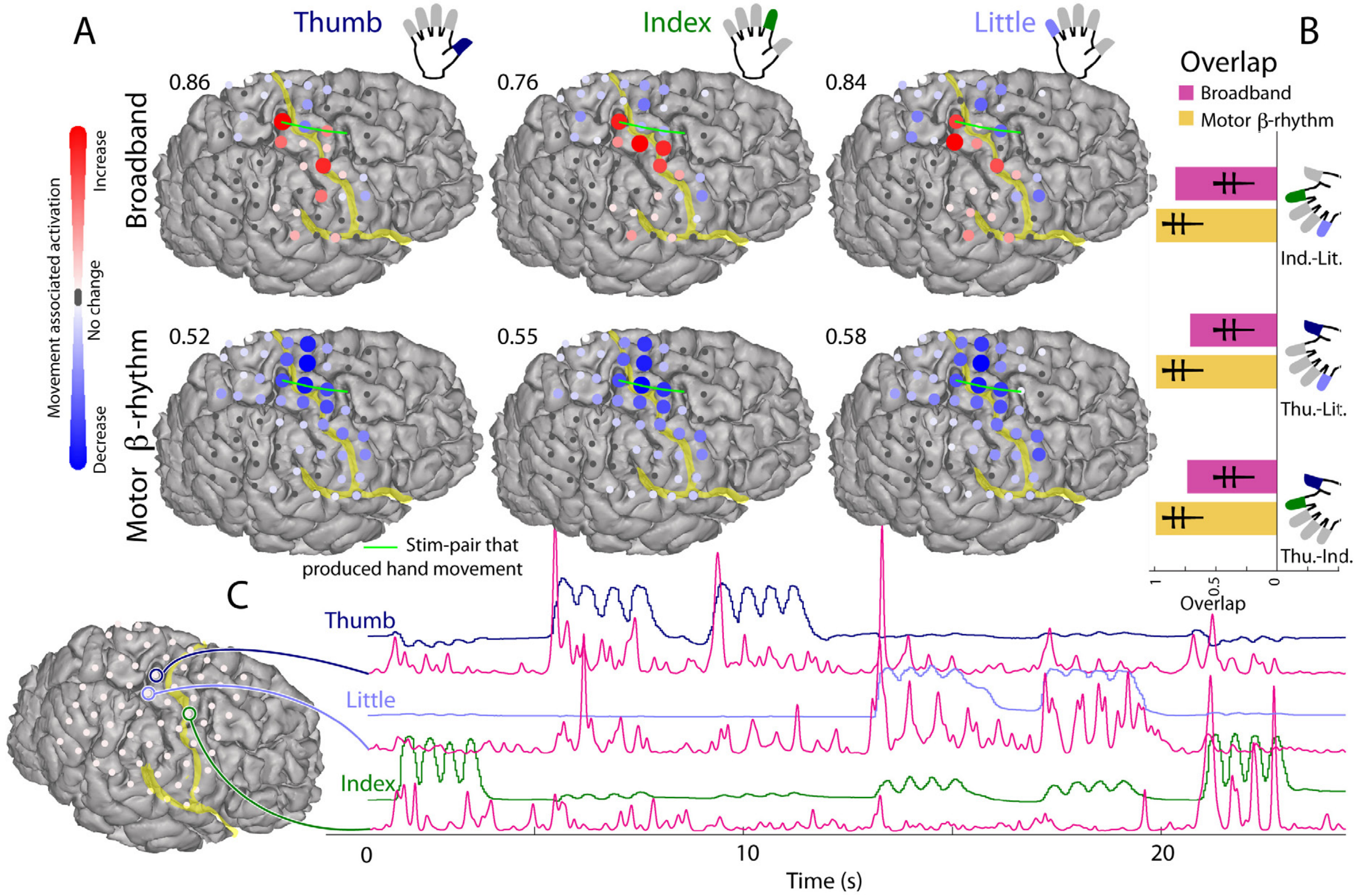

Figure S7: **Electrocorticographic finger somatotopy, for subject 2 (as in Fig 3 of the main text), but with stimulation included. Green lines indicates stimulation pairs that produces hand movement.** Electrical stimulation of the cortex to create transient lesions or induce overt movements, extra or intra-operatively, is the established method to clinically localize function in the brain [3,4,5]. This process of stimulation is critical to minimize risk in neurological surgeries which involve resection of seizure focus, tumor, or vascular malformation [6,7,8], and predicts functional outcome [8,9]. Effective mapping requires that all electrodes be stimulated with varying amounts of amperage and requires ongoing and consistent patient participation. Stimulation often leads after discharges that can induce seizures or provide misleading functional responses from distally stimulated cortex. The stimulation mapping process in the context of subdural electrode arrays is often incomplete either because it is prematurely aborted by an induced seizure, or stopped once an acceptable surgical margin is obtained. The entire array is rarely surveyed. For these reasons, many of the activation maps in this study are devoid of stimulation locations or contain incomplete stimulation information. In this sense, there are only positive stimulation results, without negative control. The only meaningful question in the context of the mapping method described here is whether the method captures a positive result. Stimulation in the context of this study was also only performed pair-wise (rather than creating a potential difference at a single electrode with respect to multiple other electrodes in the array.) In such a pair-wise regime, the cortical area proximal to a single electrode might account for the given functionality. Comparison of stimulation mapping with the spectral method here should take this into account: If the spectral method spans a single electrode from a pair, this does not imply a failure of the method, but merely illustrates an artifact of the active, pair-wise, stimulation technique used in clinical practice.

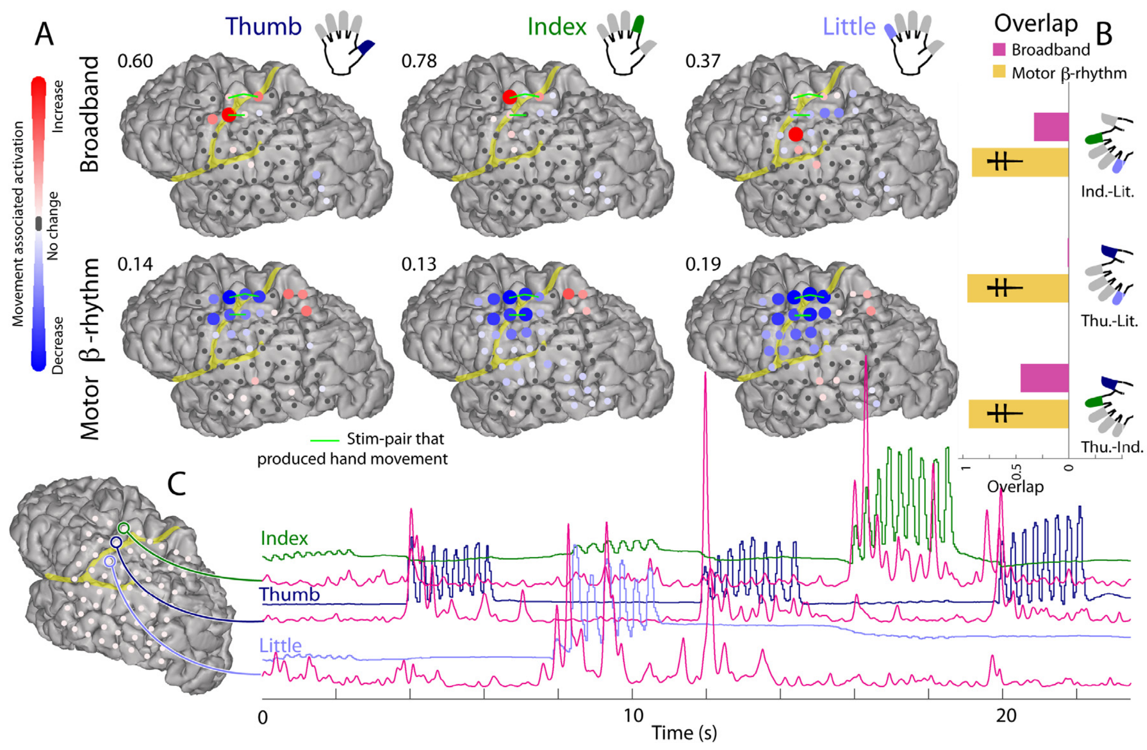

Figure S8: **Electrocorticographic finger somatotopy, subject 7, with stimulation (as in Fig S7).**

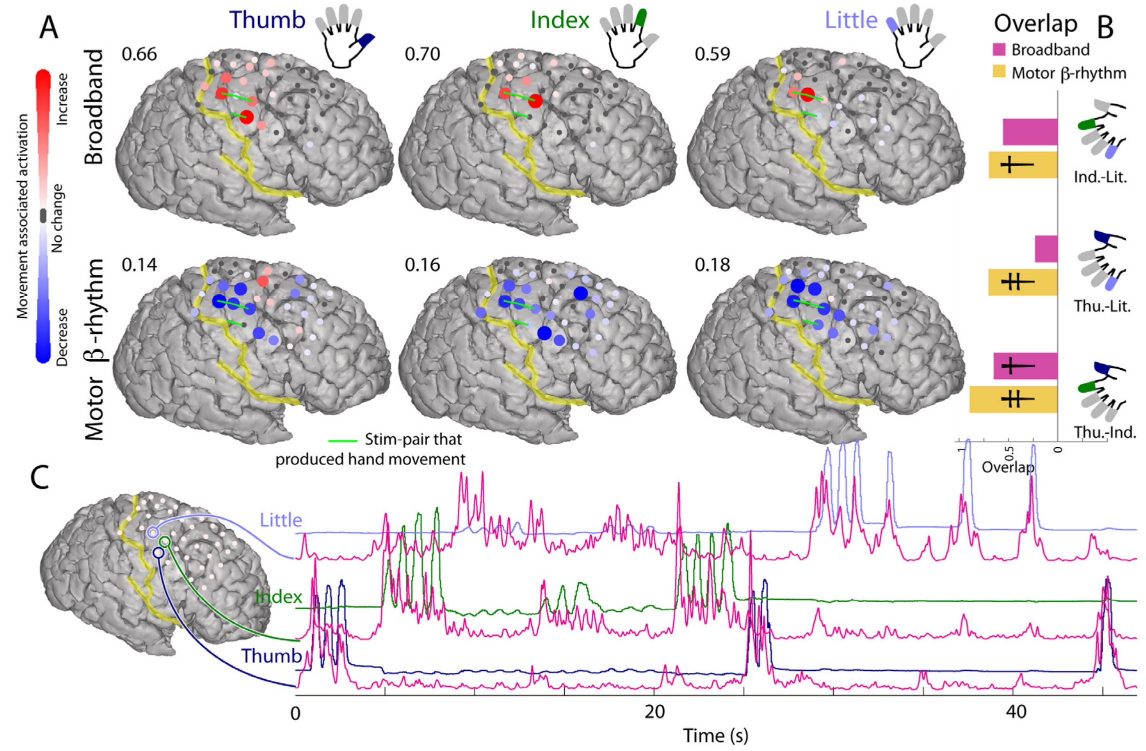

Figure S9: **Electrocorticographic finger somatotopy, subject 8, with stimulation (as in Fig S7).**

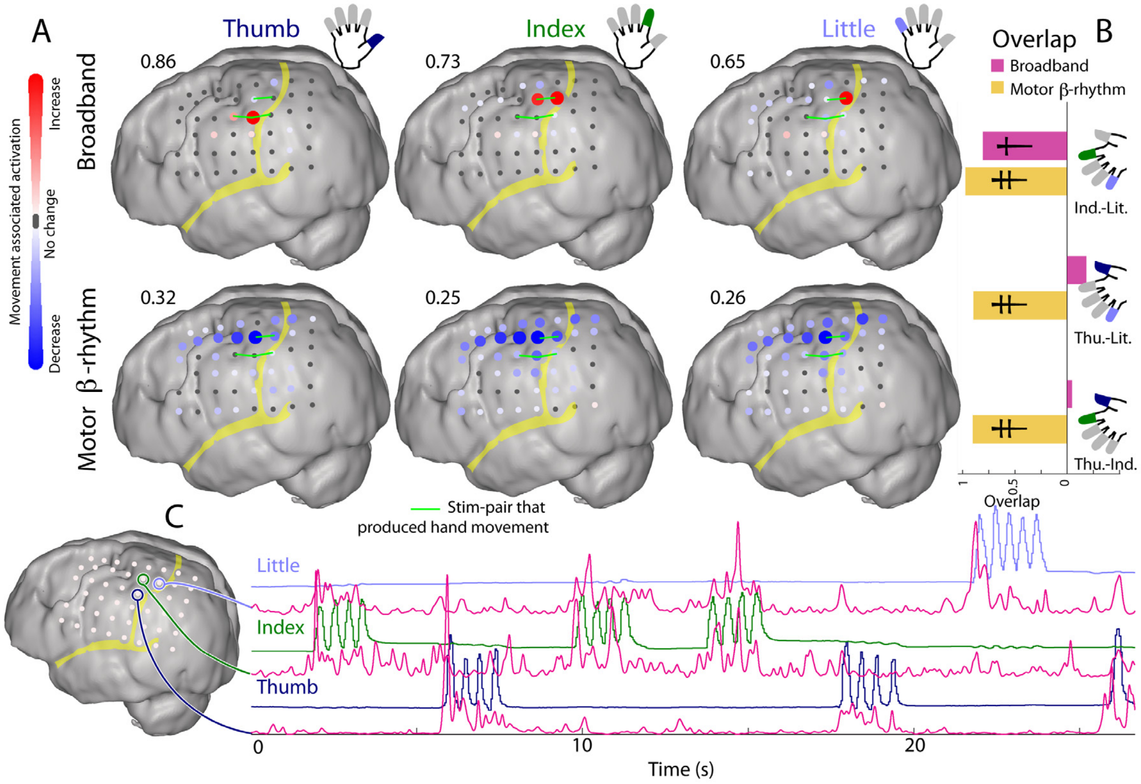

Figure S10: **Electrocorticographic finger somatotopy, subject 9, with stimulation (as in Fig S7).**

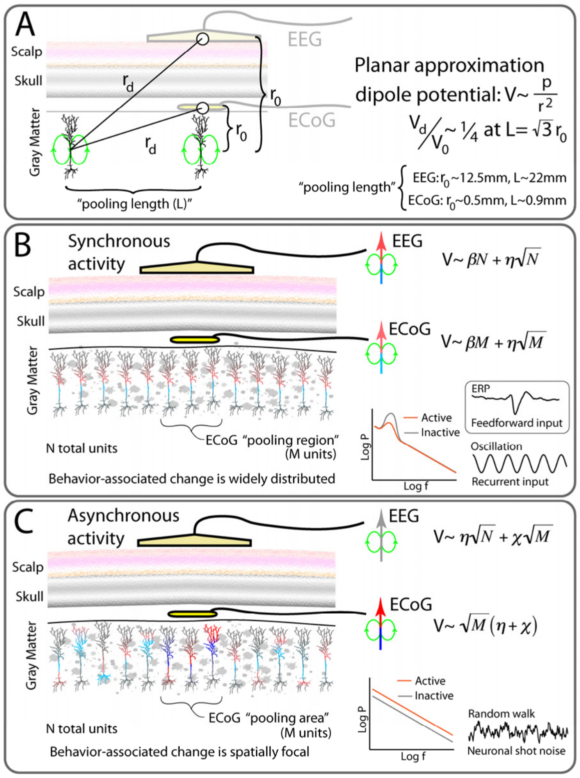

A "Back of the Envelope" comparison between EEG and ECoG

Planar approximation – We will initially ignore curvature of gyri, brain surface, skull, and scalp, and treat them all as having a stacked planar conformation.

Point measurement approximation - consider the potential only at the center of an ECoG electrode and only at the center of an EEG electrode.

Neglect effect of neuropil, tissue, skull, etc - solely consider the implications of the distance from the source of current density and the electrode.

We make a point dipole approximation for the contribution of the current source density individual pyramidal neuron to the electric potential – at EEG distance, this is appropriate, but at the ECoG distance, the quadripole moment likely contributes.

We approximate the depth of the current dipole as 0.75mm from the brain surface. The scalp surface is approximated as 12.5mm from the most superficial gyral surface.

Ignore angular dependence of dipole field

Let the depth of the dipole be approximated as roughly .5mm from gyral surface. Let scalp surface be 12.5mm from most superficial gyral surface.

We will consider the lateral extent to the limit where the contribution of a unit is 1/4 as large as the contribution directly below the electrode.

These assumptions lead us to the approximation that:

The contribution to the potential due to a single unit with dipole moment p is $V \sim p/r^2$

The perpendicular distance, L, to the furthest unit we consider ("Pooling length" - the radial distance to a unit with potential that of the closest unit) is then $L = \sqrt{3}r_0$, where r_0 is the perpendicular distance from the electrode to a dipole source directly beneath. For EEG, this corresponds to a distance of ~22mm, and for ECoG, it corresponds to a distance ~0.9mm. This corresponds to the area of pooling in EEG that is of order 500x larger than that of ECoG.

Figure S11: **The reflection of synchronous and asynchronous (broadband) phenomena in EEG and ECoG. (A)** For simplicity, we approximate the spatial extent of coherent activity of a synchronized phenomenon as being of the same order as that of the pooling region of an EEG electrode (N total units) with coefficient β, and the spatial extent of asynchrounous, broadband change, to be of the same order as that of the pooling region of an ECoG electrode (M total units). Note that "units" might refer to the dipole moment produced by input to a single synapse rather than the net dipole moment of a neuron, since macroscale field potentials are understood to reflect synaptic input. To first order, these can be treated as independent, although they will actually be correlated because of the non-linear potential dependence of active channels in the dendrite. We will approximate the sources that lie within our "pooling region" as being of equal magnitude for a 1st order approximation (although our cutoff was derived from the fact that contributions further away were of less magnitude). When considering the difference between synchronous and asynchronous cortical sources, we will also keep in mind that they will take place on top of task-independent 1/f noise of constant magnitude throughout the whole pooling range, with coefficient η. Synchronized units add linearly: $V \sim \beta N$, and asynchronous changes add in quadrature $V \sim \chi\sqrt{N}$. For *activity that is synchronized* across the whole EEG pooling region: $V \sim \beta M + \eta\sqrt{M}$ (EEG); and $V \sim \beta N + \eta\sqrt{N}$ (ECoG). For *activity that is asynchronous*, at the scale of the ECoG pooling region: $V \sim \eta\sqrt{N} + \chi\sqrt{M}$ (EEG); and $V \sim \sqrt{M}(\eta + \chi)$ (ECoG). **(B)** These approximations lead us to several conclusions about the information processing at the EEG and ECoG scales. Synchronized cortical oscillations may be differently reflected at the EEG scale than the ECoG scale. Because the influence of rhythms on local cortical activity clusters by gyral anatomy a rhythm may be more pronounced in ECoG (at the brain surface) than EEG, which averages over a spatial area (diameter 2L= ~4-5cm) that is larger than the width of a gyrus (diameter ~2cm). At the EEG scale, a rhythm might be more pronounced, due to averaging, than that of a single ECoG measurement because the synchronized portion of cortical activity adds linearly, while the background noise adds in quadrature. Likewise, a feedforward, weak-but-synchronized, event-related-potential (ERP) may be more dramatic when observed at the larger scale of EEG, where unrelated 1/f noise has been averaged away. **(C)** Broadband, 1/f, spectral changes have been demonstrated to be extremely robust correlates of local cortical activity in the ECoG signal, and are the most robust correlate of mean population firing rate at the LFP scale. These are spatially very focal, with very different behavioral specificity in adjacent electrodes that are 1cm from one another. Because these broadband shifts scale like $\chi\sqrt{N}$, even very large increases in χ will be lost at the larger EEG scale. Real neuronal populations that are measured by either ECoG or EEG will, of course, not be matched to one scale or another, but will exist in one of three regimes. They will be less than N, greater than N but less M, or greater than M. (Figure modified from [10]: Krusienski DJ, Grosse-Wentrup M, Galan F, Coyle D, Miller KJ, et al. (2011) *Critical issues in state-of-the-art brain-computer interface signal processing.* J Neural Eng 8: 025002, doi:10.1088/1741-2560/8/2/025002).

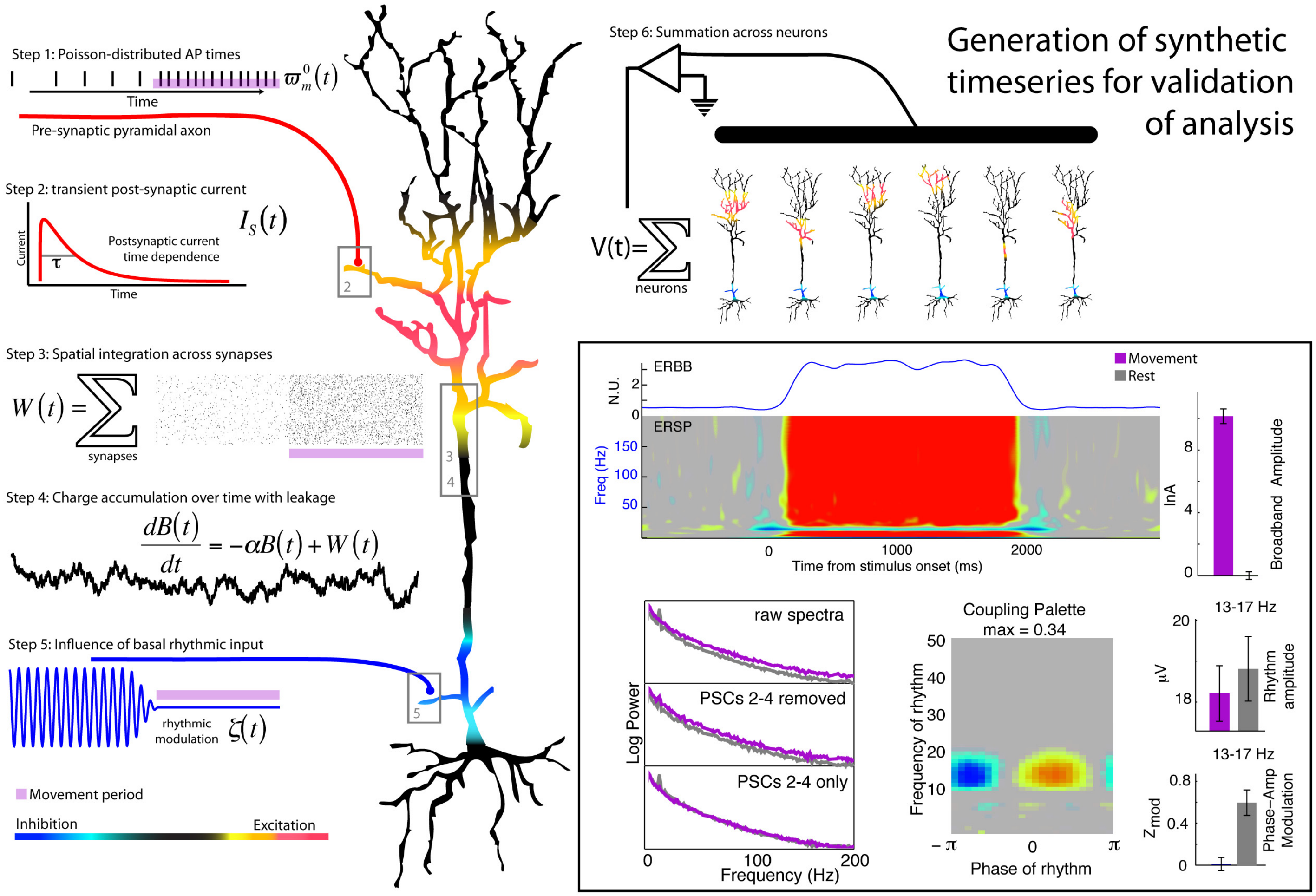

Figure S12: **Generation of a simulated timeseries and validation of signal processing technique.** The illustrated heuristic was used to generate a 1/f base, broadband, synthetic timeseries that is modulated by a 15 Hz rhythm (described in the steps below). In the boxed region on the bottom right, the synthetic timeseries is analyzed in the same way as the physiological data. It demonstrates that the decoupling and phase-modulation estimation signal processing techniques described in this manuscript work for simulated data that were based on the mechanisms hypothesized for our physiological data. *Simulation steps as follows:* ***Step 1:*** Action potentials (AP – "spikes") with Poisson-distributed inter-spike intervals arrive from a pre-synaptic cortical pyramidal neuron. The instantaneous AP rate, ϖ_m , is modulated as a function of task, so that the probability of an AP is higher during movement. ***Step 2:*** Each AP produces stereotyped transient post-synaptic current with a sharp rise, and an exponential decay of timescale $\tau_S = (2\pi 75Hz)^{-1} = 2.1ms$. The synaptic current is $I_S(t) = \eta \frac{t}{\tau_s} e^{1-t/\tau_S} \theta(t)$, where η , in this case, is a "synaptic strength" drawn randomly from [-1,1], τ_S is the time constant of the synapse, and $\theta(t)$ is a step function. ***Step 3:*** Synaptic inputs from 6000 synapses, "m", are summed at each point in time: $W(t) = \sum_m \varpi_m(t)$ and charge integrates over time. ***Step 4:*** Temporal integration and Ohmic leakage produces the broadband $B(t)$. The timecourse of $B(t)$ is determined iteratively. ***Step 5:*** A cortical rhythm $\zeta(t) = r_\zeta(t)\cos(2\pi f_0 t + \Delta(t_k))$, is simulated, with center frequency f_0 set to 15Hz. It multiplicatively modulates the broadband process, $V_{BB}(t) = (1+\zeta(t))B(t)$, and also contributes directly to the potential: $V_\zeta(t) = A_\zeta r_\zeta(t)\cos(2\pi f_0 t + \Delta(t_k) + \phi_C)$, where ϕ_c is the coupling phase (which we set to $\pi/4$), and A_ζ is the overall amplitude of the rhythmic contribution. ***Step 6:*** Simulation is performed for the summation of 10 such model neurons. A full description of this simulation process and the original illustration appear in a previous manuscript [11], and are used with permission from the author and journal.

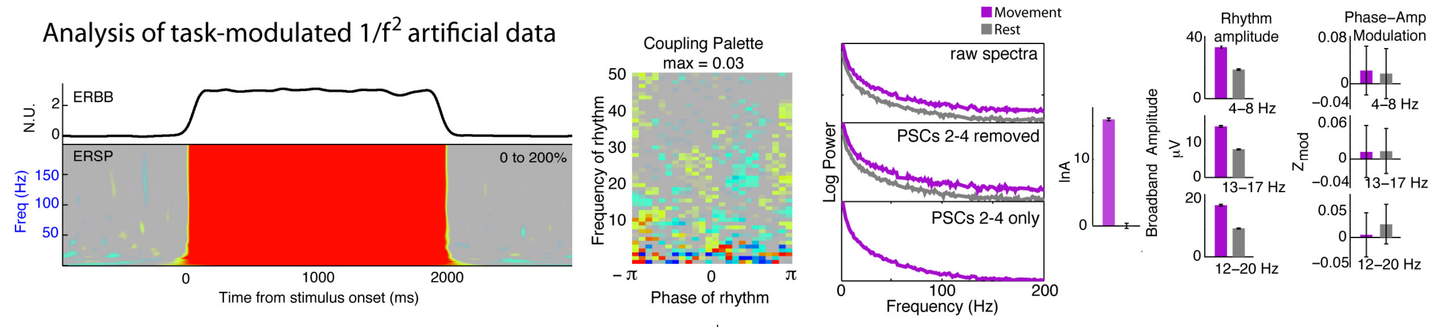

Figure S13: **Application of signal processing technique to colored noise.** When the signal processing methods were applied to task-modulated $1/f^2$ colored noise as a negative control, there is no significant coupling. A full description of this simulation process and the original illustration appear in a previous manuscript [11], and are used with permission from the author and journal.

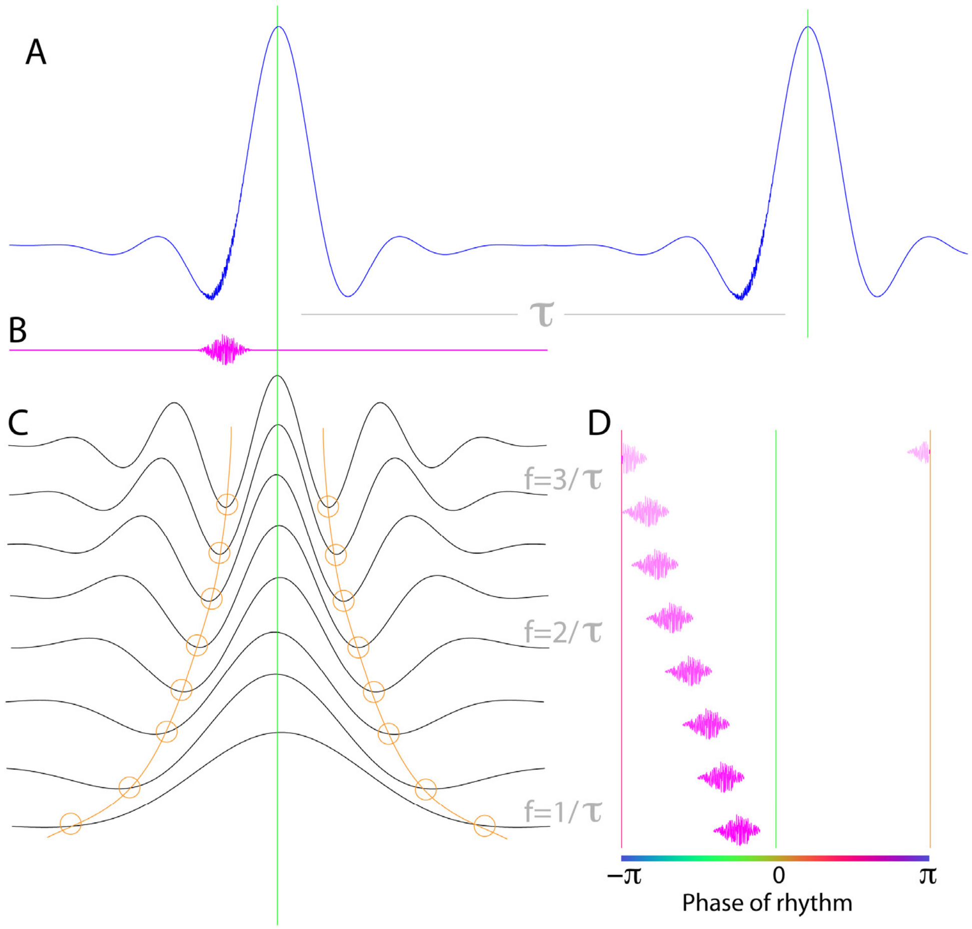

Figure S14: **A possible explanation for the diagonal bands in some coupling palettes. (A)** If the oscillations deviate from a sinusoidal form, then there may be regular-intervals of "impulses", followed or preceded by a broadband "pulse" at a fixed lag, as shown in the simulated voltage timeseries here. The principal frequency is at the inverse of the mean time between pulses. **(B)** The broadband is extracted, and there are changes at a fixed lag. **(C)** The fourier-based spectral decomposition of the raw trace reveals multiple sinusoids (as would be required to create the regular impulses). The most surface negative point of each sinusoid reveals the π phase for each (marked with an orange line for illustration). **(D)** Because the broadband "pulse" falls at a different phases for each sinusoid, the coupling palette has diagonal lines with increases in frequency.

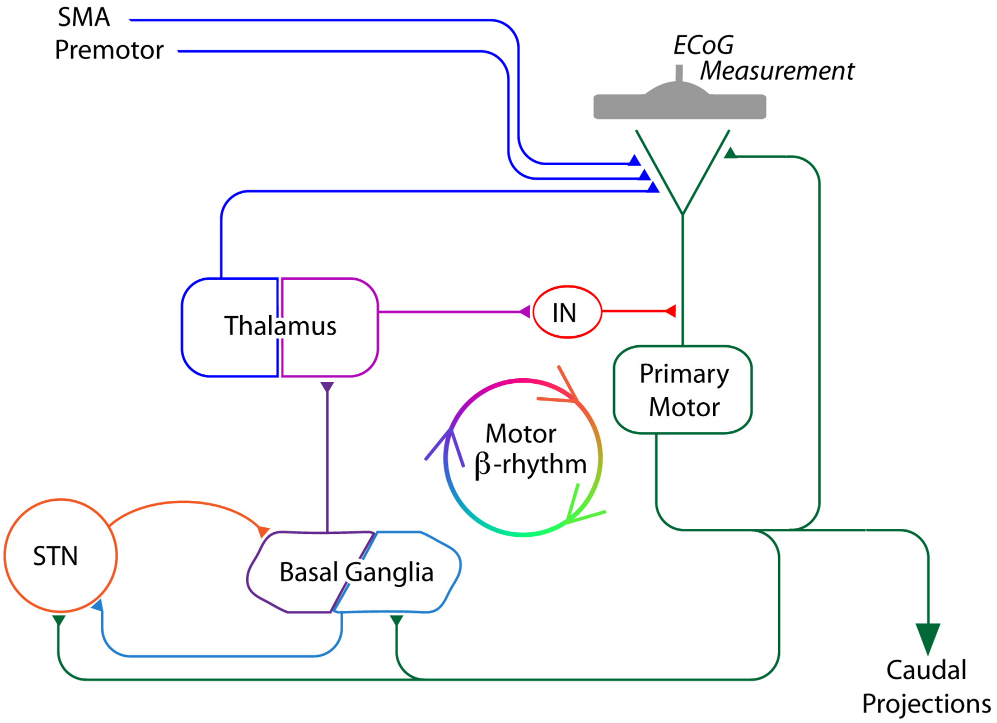

Figure S15: **A candidate large-scale mechanism for the generation of this rhythmic influence on local cortical activity.** There are many potential roles for the beta rhythm in motor cortex, with different underlying cortical mechanisms, and we hypothesize just one candidate mechanism here. The potential role we highlight is inhibition of population-scale processing by synchronization from widespread sub-cortical input to inhibitory interneurons (IN). The recurrent feedback circuit between motor cortex, basal ganglia, and thalamus (as well as input from sub-thalamic nucleus - STN) produces spatially coherent influence on pyramidal neurons in primary motor cortex. Inputs to primary motor cortex from supplemental motor area (SMA), premotor, and thalamic areas must be stochastically phase-entrained on the rhythmic influence. Focal release of this suppression through synchronization reveals how central brain structures might selectively release and engage functionally-specific regions of cortex during behavior.

4-8Hz	dorsal rol	ventral rol	frontal	parietal
ventral rol	0.031	x	1.358	4.628
frontal	0.356	1.358	x	0.050
parietal	0.000	4.628	0.050	x
temporal	4.257	0.023	0.867	0.000

Bonferroni corrected p-values

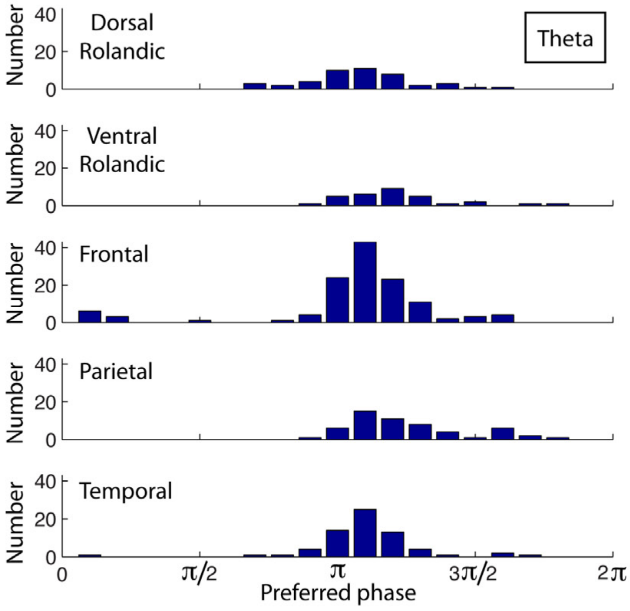

12-20Hz	dorsal rol	ventral rol	frontal	parietal
ventral rol	7.910	x	1.079	4.190
frontal	0.160	1.079	x	0.032
parietal	4.486	4.190	0.032	x
temporal	2.227	2.303	0.000	7.619

Bonferroni corrected p-values

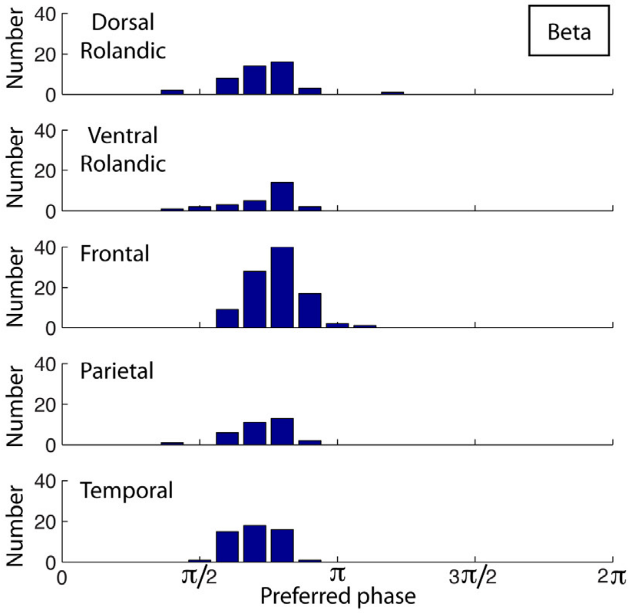

Figure S16: **Preferred phase of modulation by anatomic region, pooled across all subjects.** ***On the left (Theta, 4-8Hz),*** all electrodes that showed significant modulation of broadband activity by 4-8Hz phase in the fixation task were pooled for analysis (e.g. subjects 1-4, 7-12, as in figure 7 of the main text, with the same anatomic delineations). In the ***table at the top***, the comparison of preferred phase of coupling was performed pairwise for all electrodes in each of two areas (across subjects) as follows: The mean coupling vectors are calculated for region 1 and region 2 independently – the true difference in coupling phase is the absolute value of the difference in the preferred phase of these two. Then, all electrodes of these two groups are pooled, and randomly divided into two groups of the same sizes as region 1 and region 2, and a surrogate difference in phase is generated. This is done 10^4 times to generate a surrogate distribution. The raw p-value to determine if there is a difference in preferred phase is then the proportion of surrogate preferred phase differences that is larger than the true difference in coupling phase. The table shows these raw p-values multiplied by 10 (a Bonferroni correction for the number of pair-wise region comparisons made). *For theta (4-8 Hz), this suggests that there is a statistically significant ($p<0.05$) difference in preferred phase of coupling between 1) dorsal rolandic cortex and ventral rolandic cortex (rolandic = pre- & post-central), 2) dorsal rolandic cortex and parietal cortex (parietal cortex excluding post-central gyrus), 3) ventral rolandic cortex and temporal cortex, 4) frontal cortex and parietal cortex (excluding pre- & post-central gyri), and 5) temporal and parietal cortex.* The ***histograms below the table*** show the distributions of preferred coupling phases by brain region. ***On the right (Beta, 12-20Hz),*** all electrodes that showed significant modulation of broadband activity by 12-20Hz phase in the fixation task were pooled for analysis. *For (Beta 12-20 Hz), this suggests that there is a statistically significant ($p<0.05$) difference in preferred phase of coupling between 1) frontal cortex and parietal cortex (excluding pre- & post-central gyri), and 2) frontal and temporal cortex.*

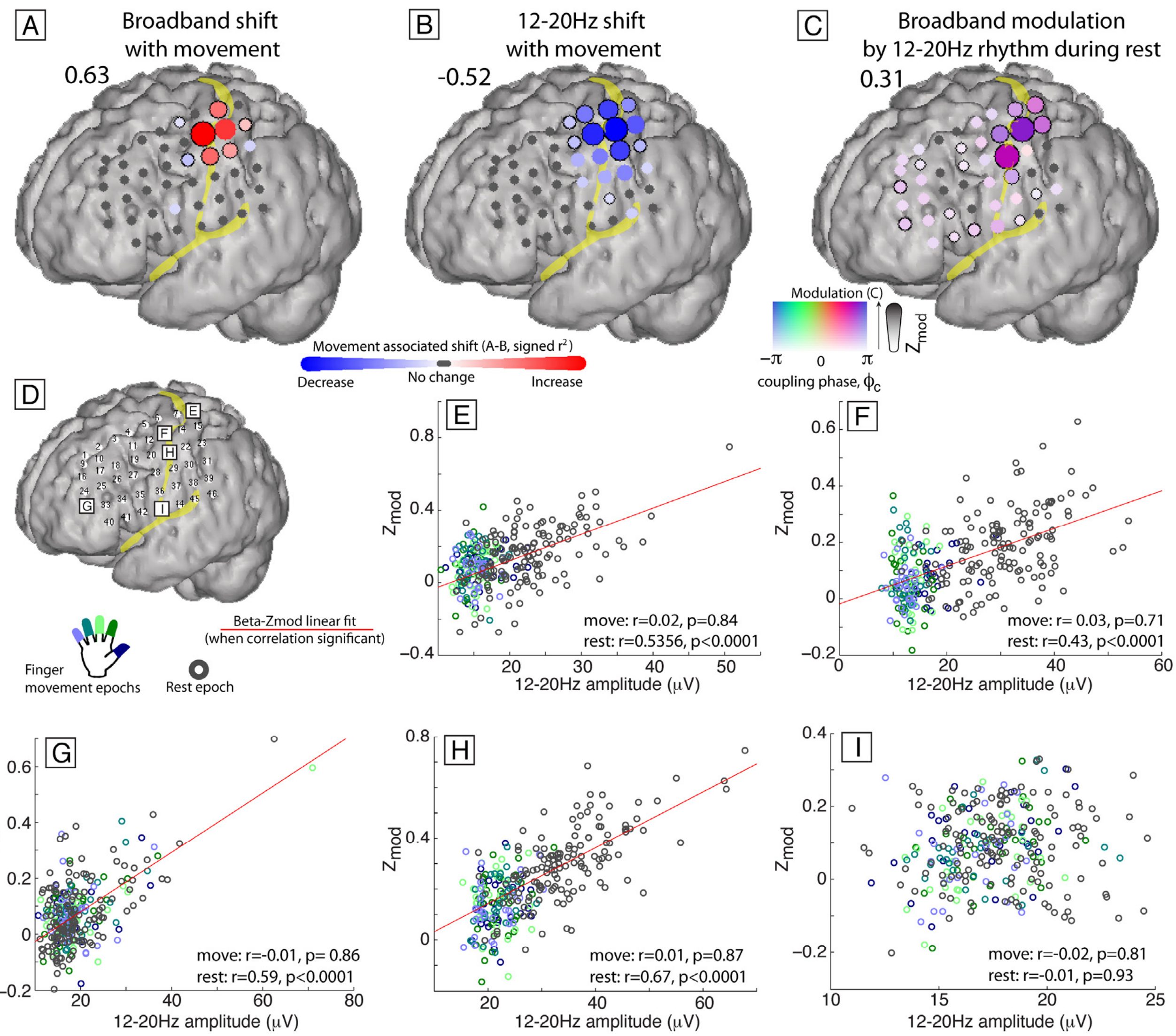

Figure S17: **The conditional relationship between broadband, beta (β, 12-20Hz), and modulation.** The structural relationship between modulation of local activity by the β-rhythm and β-amplitude, during movement and rest is not simple. An interesting issue posed by these experiments is what the basic relationship is (if any) between the amplitude of the β-rhythm and the coincident degree of modulation is. **(A)** The spatial distribution of sites showing a broadband spectral change associated with index finger movement, subject 1. (color represents a signed r^2 measurement, scaled to the maximum across the array: 0.63). In these figures the Central sulcus and Sylvian fissure are shown in yellow.Broadband spectral changes associated with thumb movement are similarly shown (maximum: 0.64). **(B)** The spatial distribution of sites showing a decrease in 12-20Hz power associated with index finger movement, subject 1. **(C)** 12-20Hz modulation in the grid, subject 1. The strength of color and diameter denote the magnitude of coupling, and the color denotes the preferred phase of coupling.

These spatial maps beg the questions (or, at least did for an astute reviewer of this manuscript!): “What is the causal structure between 1) β-broadband modulation (Z_{mod}), 2) β-amplitude, and 3) movement? Specifically, does movement influence PAC directly, or by using beta power as an intermediate variable?” **(D)** As such, we illustrate the relationship between beta-broadband modulation for subject 1 at a variety of sites, noted E-I. **(E)** Modulation as a function of beta amplitude is shown, sorted by movement (colors), and rest (gray). There is a significant correlation between these during rest (red line shows fit line for this), but not during movement periods (all movements combined as a group). This phenomenon was true at many **(E-H)**, but not all **(I)**, sites.

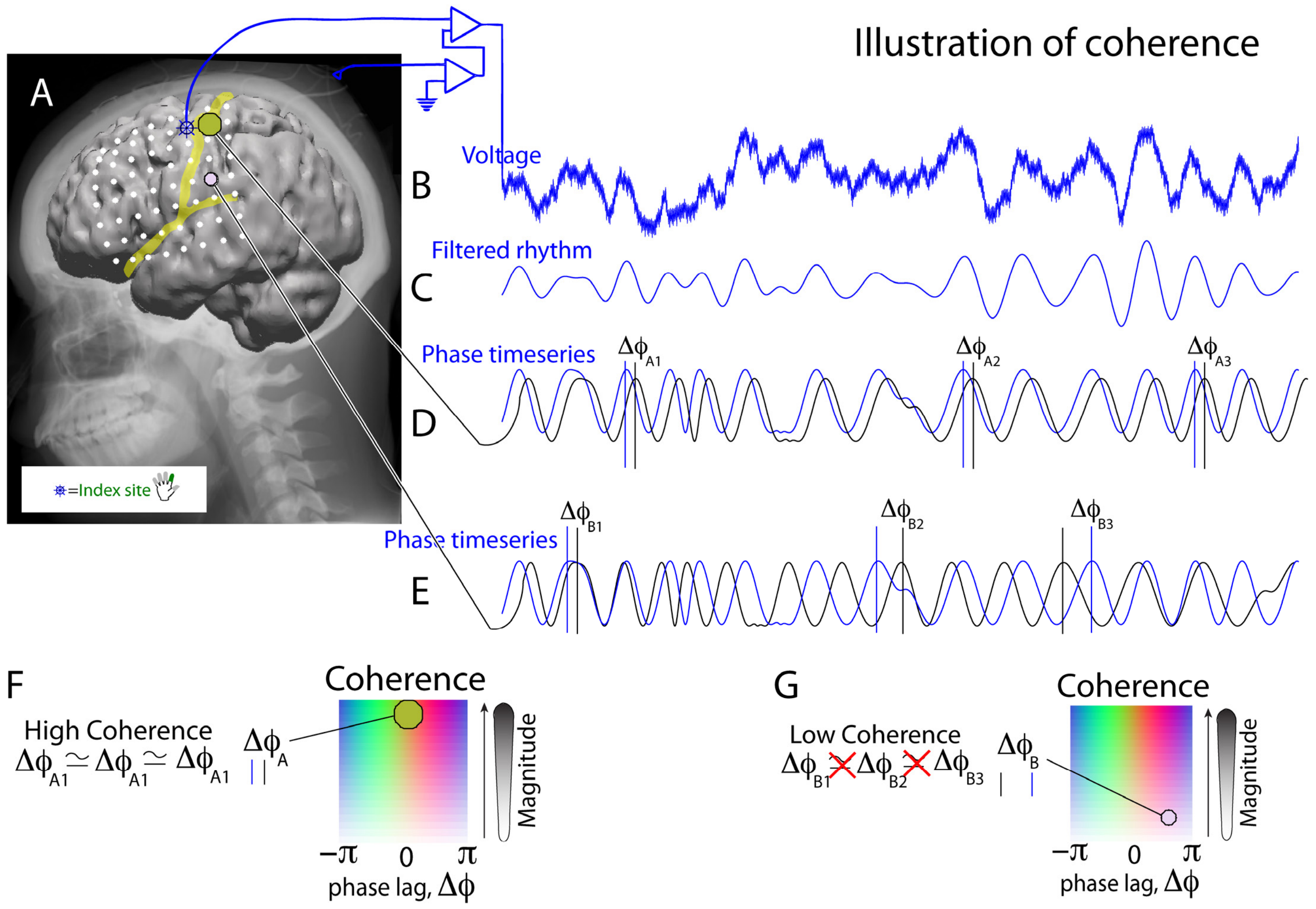

Figure S18: **Illustration of pair-wise phase coherence relative to a reference "seed" site.** **(A)** The electrode array is shown in situ. **(B)** The voltage timeseries $V_a(t)$ from a seed site, the motor electrode with the strongest index finger movement-associated broadband change, is compared with all other sites. **(C)** The voltage timeseries from each site is band-pass filtered for the particular frequency range of interest, to obtain $V_a(F,t)$. **(D)** The Hilbert transform of $V_a(F,t)$, $V_a^H(F,t)$, is obtained, and the "complex rhythm" $\tilde{V}_a(F,t) = V_a(F,t) + iV_a^H(F,t)$ is constructed. This can be represented in polar notation as $\tilde{V}_a(F,t) = r_a(F,t)e^{i\phi_a(F,t)}$. The Hilbert phase of this timeseries, $\phi_a(F,t)$, from each electrode is used to calculate phase coherence. In (D), we illustrate the oscillation of this phase with $\cos(\phi_a(F,t))$. Phase coherence with another site that has high coherence, and a small phase lag. Although the actual quantity for the coherence is $Q_{ab}(F) = \frac{1}{T}\sum_t e^{i(\phi_a(F,t)-\phi_b(F,t))}$, $\cos(\phi_a(F,t))$ and $\cos(\phi_b(F,t))$ are shown for illustration. If the phases have a consistent lag with respect to one another over time, the phase coherence will be high. How this is plotted is illustrated in (F). **(E&G)** As in (D&F), but for a site with low coherence and a large phase lag. Note that in D, the illustration shows a variable phase lag between the two.

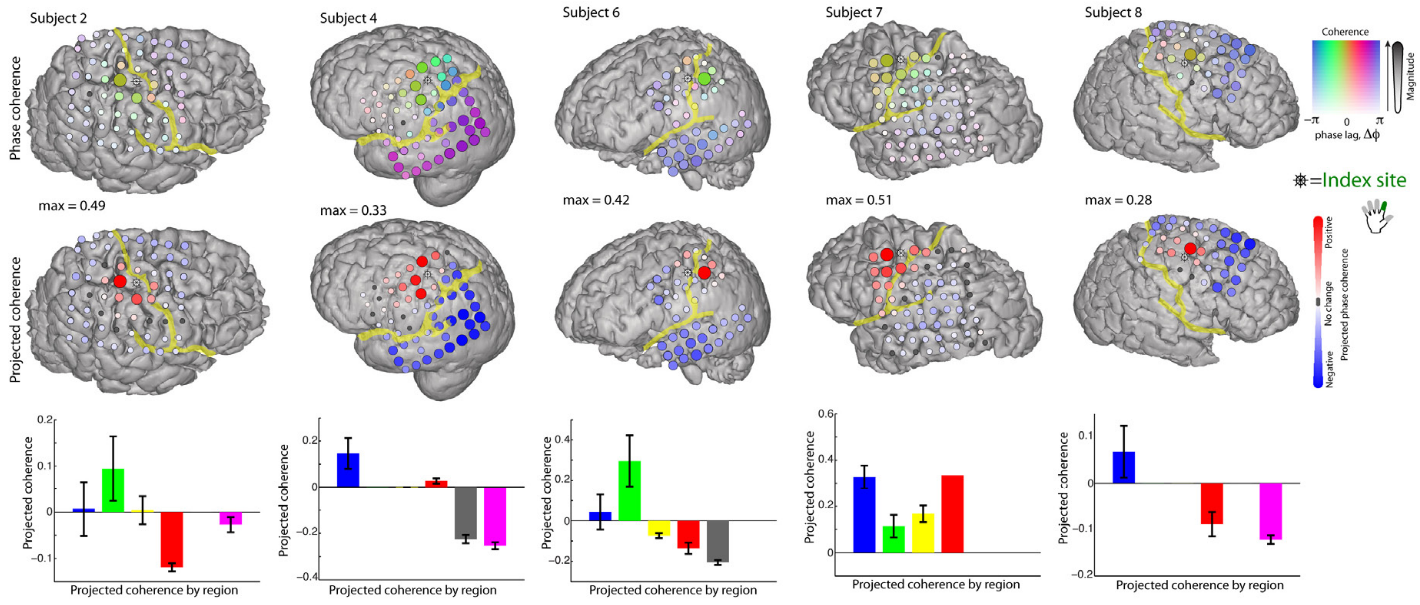

Figure S19: **Pair-wise 12-20Hz coherence with a peri-central index-finger specific electrode across subjects.** As in figures 11 and 12, for subjects 2, 4, 6, 7, and 8.

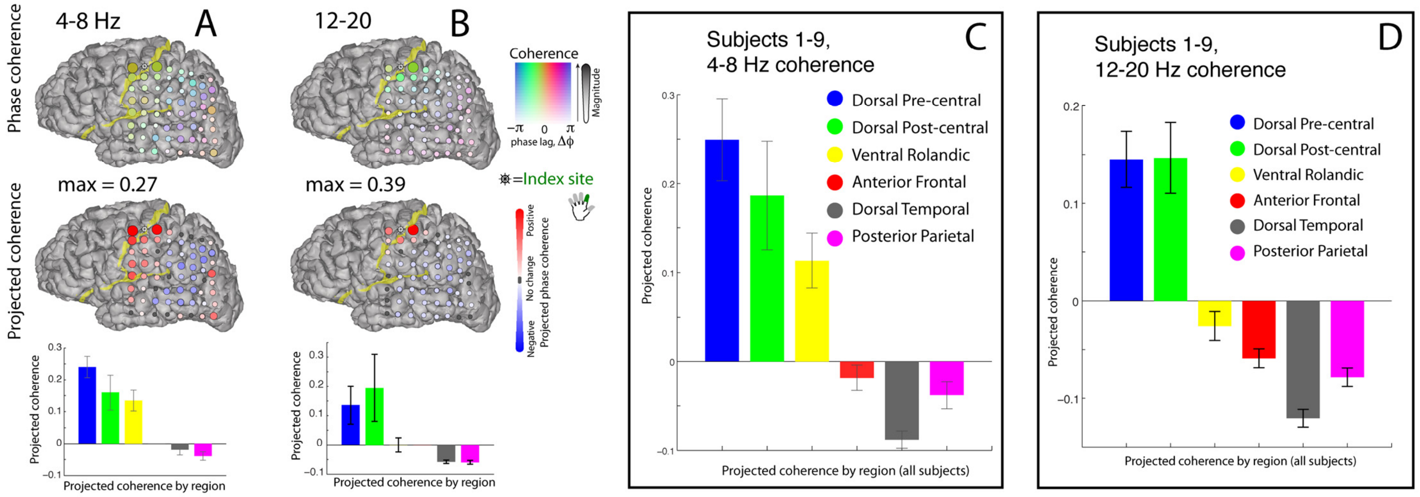

Figure S20: **A comparison of coherence with an index finger "seed site" for the theta (4-8Hz) and beta (12-20Hz) ranges. (A)** as in figures 11, 12, S19, but for subject 5, and for 4-8Hz. Note that the coherence still obeys gyral anatomy, including the dorsal and ventral rolandic areas. **(B)** Subject 5, coherence for 12-20Hz. Note that coherence is focused in dorsal rolandic cortex. **(C)** 4-8Hz coherence, across subjects 1-9. Note that the theta rhythm is coherent across both dorsal and ventral rolandic areas. **(D)** 12-20Hz coherence, across subjects 1-9. Note that the beta rhythm is coherent across dorsal rolandic cortex. This illustrates that both the theta and beta rhythm coherences obey gyral anatomy, but it is different in distribution for the two rhythms.

Text S1, Supplement to: Human motor cortical activity is selectively phase-entrained on underlying rhythms,

Subject	Age	Sex	Handedness	Array location
1	18	F	R	L Frontoparietal
2	21	M	R	R Frontotemporal
3	27	F	R	L Fronto-temporal-parietal
4	35	F	R	L Fronto-Temporal
5	26	M	R	L Parietal - Temporal - Occipital
6	45	F	R	L Frontotemporal
7	32	M	R	L Fronto-temporal-parietal
8	19	F	R	R Fronto-parietal
9	18	F	R	L Frontal
10	42	M	R	L Frontotemporal
11	41	M	L	L Frontotemporal
12	39	F	R	R Frontal
13	13	M	R	L Fronto-parietal
14	17	F	R	R Fronto-parietal

Supplemental Table: **Patient characteristics**

Supplemental references:

1. Osipova D, Hermes D, Jensen O (2008) Gamma power is phase-locked to posterior alpha activity. PLoS One 3: e3990.
2. Canolty RT, Edwards E, Dalal SS, Soltani M, Nagarajan SS, et al. (2006) High gamma power is phase-locked to theta oscillations in human neocortex. Science 313: 1626.
3. Ojemann G, Ojemann J, Lettich E, Berger M (1989) Cortical language localization in left, dominant hemisphere. An electrical stimulation mapping investigation in 117 patients. J Neurosurg 71: 316-326.
4. Chitoku S, Otsubo H, Harada Y, Jay V, Rutka JT, et al. (2001) Extraoperative cortical stimulation of motor function in children. Pediatr Neurol 24: 344-350.
5. Branco DM, Coelho TM, Branco BM, Schmidt L, Calcagnotto ME, et al. (2003) Functional variability of the human cortical motor map: electrical stimulation findings in perirolandic epilepsy surgery. J Clin Neurophysiol 20: 17-25.
6. Berger MS, Kincaid J, Ojemann GA, Lettich E (1989) Brain mapping techniques to maximize resection, safety, and seizure control in children with brain tumors. Neurosurgery 25: 786-792.
7. Burchiel KJ, Clarke H, Ojemann GA, Dacey RG, Winn HR (1989) Use of stimulation mapping and corticography in the excision of arteriovenous malformations in sensorimotor and language-related neocortex. Neurosurgery 24: 322-327.
8. Haglund MM, Berger MS, Shamseldin M, Lettich E, Ojemann GA (1994) Cortical localization of temporal lobe language sites in patients with gliomas. Neurosurgery 34: 567-576; discussion 576.
9. Keles GE, Lundin DA, Lamborn KR, Chang EF, Ojemann G, et al. (2004) Intraoperative subcortical stimulation mapping for hemispherical perirolandic gliomas located within or adjacent to the descending motor pathways: evaluation of morbidity and assessment of functional outcome in 294 patients. J Neurosurg 100: 369-375.
10. Krusienski DJ, Grosse-Wentrup M, Galan F, Coyle D, Miller KJ, et al. (2011) Critical issues in state-of-the-art brain-computer interface signal processing. J Neural Eng 8: 025002.
11. Miller KJ, Hermes D, Honey CJ, Sharma M, Rao RP, et al. (2010) Dynamic modulation of local population activity by rhythm phase in human occipital cortex during a visual search task. Front Hum Neurosci 4: 197.
